# Molecular Structure of Foldable Bottlebrush Polymers in Melts

**DOI:** 10.1021/acs.macromol.4c02981

**Published:** 2025-04-01

**Authors:** Li-Heng Cai

**Affiliations:** † Soft Biomatter Laboratory, Department of Materials Science and Engineering, University of Virginia, Charlottesville, Virginia 22904, United States; ‡ Department of Chemical Engineering, University of Virginia, Charlottesville, Virginia 22904, United States; § Department of Biomedical Engineering, University of Virginia, Charlottesville, Virginia 22904, United States; ∥ Department of Chemistry, University of Virginia, Charlottesville, Virginia 22904, United States

## Abstract

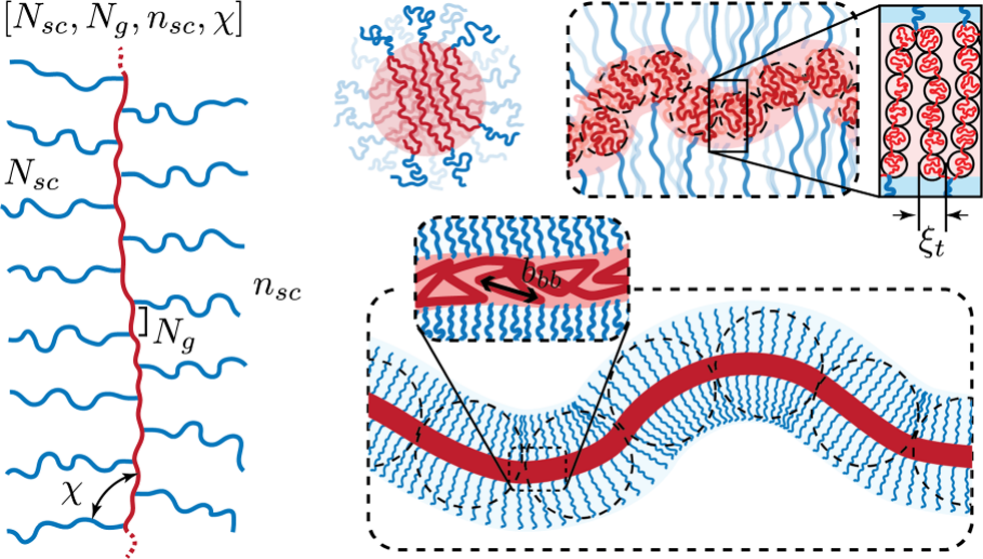

A bottlebrush polymer consists of a long linear backbone densely grafted with many relatively short side chains. A widely accepted view is that strong steric repulsion among the highly overlapped side chains prestrains the bottlebrush backbone, resulting in low polymer extensibility. However, we recently discovered that in the melt of bottlebrush polymers with highly incompatible side chains and backbone, the backbone collapses to reduce interfacial free energy, regardless of the strong steric repulsion among side chains. Despite this discovery, the molecular structure of these so-called “foldable” bottlebrush polymers and their assemblies remains poorly understood. Here, we present the deterministic relationships among molecular architecture, mesoscopic conformation, and macroscopic properties of foldable bottlebrush polymers. A combination of scaling theory and experiments reveals that as the side chain grafting density decreases, the bottlebrush diameter increases, whereas the bottlebrush end-to-end distance decreases. These behaviors contradict the existing understanding of bottlebrush polymers, which assumes that the backbone and side chains are compatible. Since foldable bottlebrush polymers store lengths that can be released upon large deformations, they offer a way to decouple the intrinsic stiffness-extensibility trade-off in single-network elastomers. These findings provide foundational insights into using foldable bottlebrush polymers as building blocks for designing soft (bio)materials.

## 1 Introduction

A bottlebrush polymer consists of a long linear backbone densely grafted with many relatively short linear side chains. It can either be synthetically made through controlled polymerization techniques^1−3^ or exist as natural biopolymers such as aggrecan^4−8^ and mucins in biological systems.^9−11^ Analogous to sausage, as opposed to spaghetti, a bottlebrush polymer is essentially a “fat” linear polymer that is difficult to entangle, enabling entanglement-free, extremely soft polymer networks with prescribed tissue-mimicking stiffness.^12−19^ Additionally, bottlebrush molecules can be tuned in size from nanometers^20,21^ to micrometers^23−29^ to create structures with mesoscale characteristic lengths and multiscale ordering, enabling soft materials of fascinating rheological, mechanical, optical, and dielectric properties;^23−29^ examples include bottlebrush polymer-based super lubricants,^31−34^ adhesives,^31−34^ photonic crystals,^35−37^ and dielectric elastomer actuators.^26^ Moreover, the steric repulsion among overlapping side chains prestrains the bottlebrush backbone, and the extent of prestretching can be prescribed by the grafting density and/or the size of side chains to match the strain-stiffening behavior of various biological tissues.^38^ Further, constituent side chains can be functionalized to achieve tissue-specific biochemical properties without impairing the physical properties of the bottlebrush polymer. For instance, bottlebrush polymers can be used as drug carriers^39−48^ and contrast agents for in vivo imaging.^49^ Thus, mechanical, physical, and biochemical complexities can be independently encoded into the molecular architecture of bottlebrush molecules. Yet, as for classical linear polymers, using bottlebrush polymers as building blocks to create functional materials requires understanding the deterministic relation between their molecular structure and architectural parameters.^22,30,58,61−63^

In the melt, the molecular structure of a bottlebrush polymer is largely determined by how to pack the side chains within a limited space surrounding the bottlebrush backbone. Unlike melts of linear polymers where individual chains are free to move, in a bottlebrush molecule the side chains are covalently linked to the bottlebrush backbone. These side chains are highly overlapping with each other, such that they must stretch radially away from the bottlebrush backbone to avoid crowding. In doing so, the side chains occupy a cylindrical volume centering the contour of the bottlebrush backbone. Thus, a bottlebrush polymer can be treated as a semiflexible, wormlike linear polymer with a renormalized Kuhn segment size about the bottlebrush diameter. Because Kuhn segments are space-filling in the melt, the bottlebrush diameter almost equals the interbackbone distance of two neighboring bottlebrush polymers. As the grafting density decreases, the steric repulsion among side chains is alleviated, such that the side chains become less stretched. As a result, it is widely accepted that the bottlebrush diameter decreases with the decrease of the side chain grafting density, as documented in a seminal work by Rubinstein Lab.^50^

Recently, we experimentally discovered that in the melt of bottlebrush polymers, the bottlebrush diameter, or the interbackbone distance, increases monotonically with the decrease of side chain grafting density,^51^ a phenomenon contrary to the widely accepted view of bottlebrush polymers. We reasoned that this remarkable phenomenon is attributed to the incompatibility between the side chains and the bottlebrush backbone. Despite the strong steric repulsion among the highly overlapped side chains, the bottlebrush backbone folds into a cylindrical core with all grafting sites on its surface to reduce interfacial free energy. As the grafting density decreases, the backbone polymer collapses; this process not only increases the diameter of the cylindrical core but also reduces the distance between grafting sites in space, such that the extension of side chains is not alleviated. Contrary to conventional bottlebrush (cBB) polymers in which the bottlebrush backbone is prestretched, this so-called foldable bottlebrush (fBB) polymer stores lengths in the collapsed bottlebrush backbone. Upon elongation, the collapsed backbone unfolds to release stored length, enabling remarkable extensibility. By contrast, the molecular weight (MW) of fBB polymer is dominated by the side chains. As a result, using fBB polymers as network strands provides a universal strategy to decouple the inherent stiffness-extensibility trade-off of single-network elastomers.^52^ These discoveries highlight the potential of fBB polymers as a novel platform for soft (bio)materials design and innovation. Yet, the molecular structure of fBB polymers and their assemblies remains to be understood.

In this work, we present a scaling theory for the molecular structure of fBB polymers in the melt. The paper is structured as follows. In Section 2, we introduce the molecular architecture parameters of a grafted polymer. In Section 3, we summarize the prevailing understanding of the molecular structure of cBB polymers, in which the side chains and backbone are assumed to be compatible. In Section 4, we present the theory for fBB polymers consisting of highly incompatible side chains and backbone. We also comment on the segregation strength below which the incompatibility between the side chains and the backbone is not strong enough to result in collapsed bottlebrush backbone. In Section 5, we discuss the difference in experimentally measurable physical properties (diameter and extensibility) for cBB and fBB polymers. In Section 6, we compare theoretical predictions with experiments. Finally, we summarize the characteristics of fBB polymers, discuss their implications, and comment on open questions. A list of symbols is provided at the end of the paper.

## 2 Molecular Architecture Parameters of a Grafted Polymer

**Figure 1:**
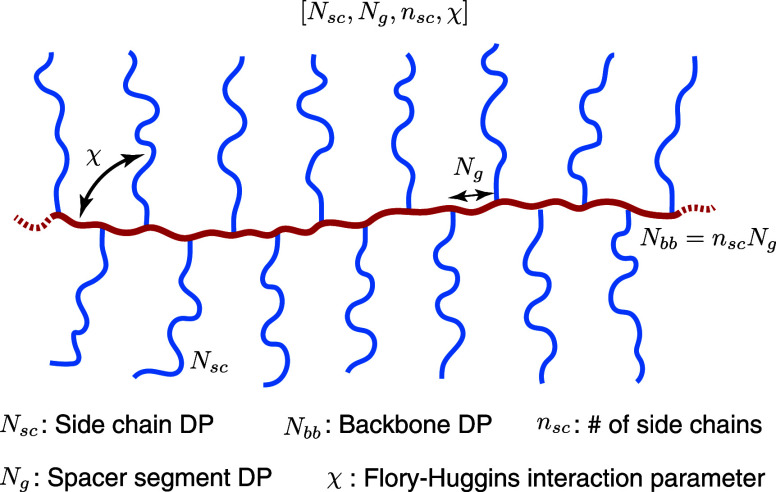
Molecular architecture parameters of a grafted polymer. A grafted polymer consists of a long linear backbone grafted with many (*n*_sc_) relatively short side chains. The degree of polymerization (DP), or the number of chemical monomers, of the side chain, the average DP of the spacer segment between two neighboring grafting sites, and the DP of the bottlebrush backbone are denoted as *N*_sc_, *N*_g_, and *N*_bb_ = *n*_sc_*N*_g_, respectively. The Flory–Huggins interaction parameter between the bottlebrush backbone (red line) and the side chains (blue lines) is denoted as χ. Thus, for a grafted polymer there are four molecular design parameters, [*N*_sc_, *N*_g_, *n*_sc_, χ].

We consider a grafted polymer consisting of a long linear backbone grafted by many relatively short linear side chains, as illustrated in Figure 1. The degree of polymerization (DP) of a side chain is denoted as *N*_sc_, and the average DP of the spacer segment between two neighboring grafting sites is denoted as *N*_g_. The number of side chains per grafted polymer, *n*_sc_, is much larger than the DP of the side chain, *n*_sc_ ≫ *N*_sc_, such that the effects of extra space near the two ends of a grafted polymer on the polymer conformation can be ignored. We use *l*, *v*, *b*, *v*_K_, *L*_max_, respectively, to denote the length of the main-chain bonds of a chemical monomer, the volume of a chemical monomer, the length of a Kuhn segment, the volume of a Kuhn segment, and the contour length of a linear polymer.

In most experiments, the side chains are not necessarily of the same chemical species as the backbone. Thus, often the side chains and the backbone are incompatible with a Flory–Huggins interaction parameter χ > 0. To this end, we use “sc” and “bb” as subscripts or superscripts to denote side chains and backbone, respectively. Additionally, we provide simplified expressions that disregard the difference in polymer physics parameters between the side chains and the bottlebrush backbone. This simplification aids in distilling the essential physical pictures for the molecular structure of bottlebrush polymers.

## 3 Conventional Bottlebrush Polymers with Compatible Backbone and Side Chains

In a cBB polymer, the side chains and the backbone are assumed to be compatible (χ = 0). An example is a poly(dimethylsiloxane) (PDMS) bottlebrush in which the bottlebrush backbone and the side chains are both linear PDMS, as exemplified in our previous work.^13^ The conformations of constituent side chains and backbone are determined by the minimization of entropic free energy. However, the side chains are not free; instead, they are constrained in space near the bottlebrush backbone. Consequently, the molecular structure of a conventional grafted polymer is determined by the packing of side chains in a limited space. Depending on the grafting density of side chains, the molecular structure of the grafted polymer can be classified into *four* regimes.^50^

**Figure 2:**
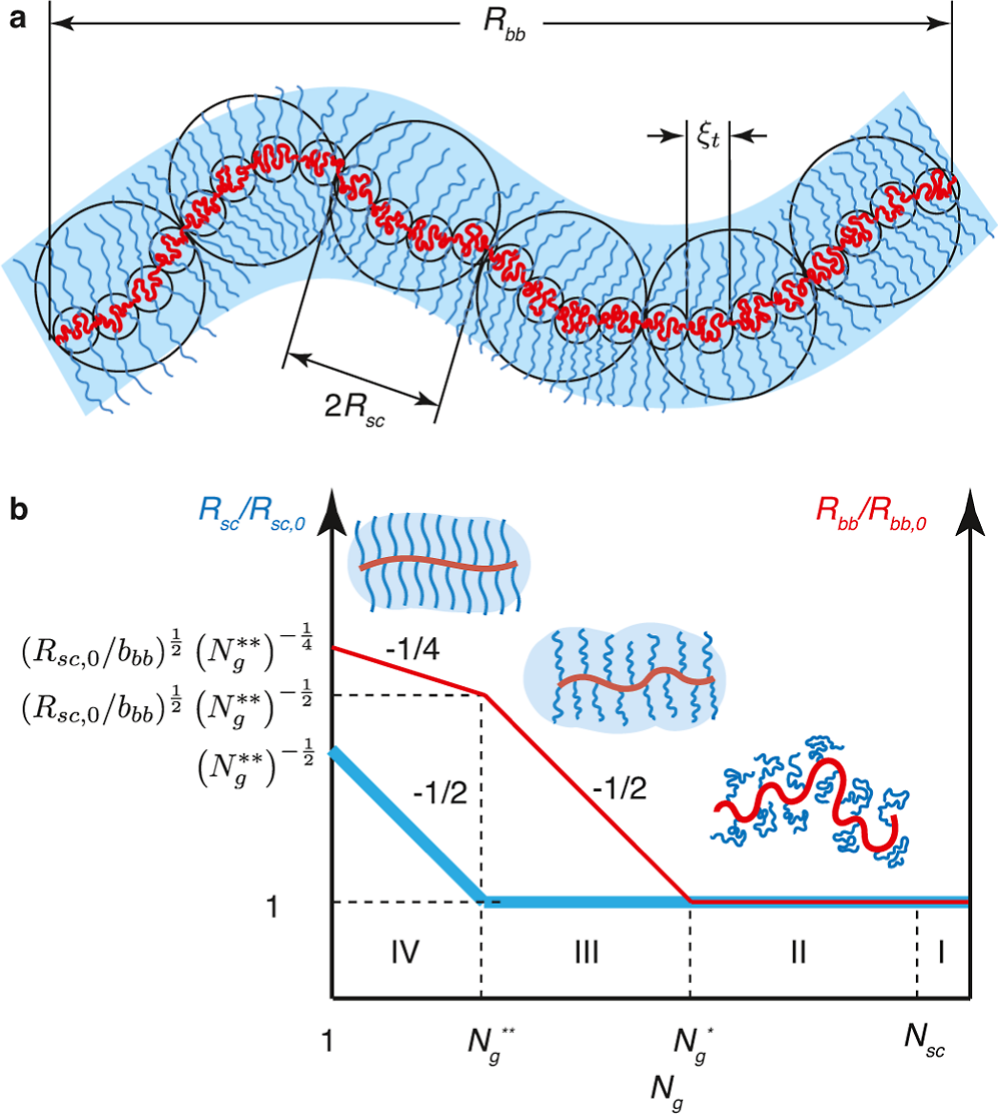
Molecular structure of a conventional bottlebrush polymer in the melt. In a conventional bottlebrush polymer, the side chains and the bottlebrush backbone are assumed to be compatible (χ = 0). (a) A schematic for the conformation of a bottlebrush polymer. The mean-square end-to-end distance of the bottlebrush backbone, *R*_bb_, is a random walk of effective monomers (large, black circles) with the persistence length about the size of a side chain, *R*_sc_. At length scales smaller than a persistence length but larger than the tension blob size ξ_t_, the backbone is a stretched array of tension blobs. At length scales smaller than ξ_t_, the backbone is a random walk of Kuhn segments of size *b*_bb_. (b) Scaling regimes for the relative increase of sizes for the side chain, *R*_sc_/*R*_sc,0_ (thick blue lines), and the bottlebrush backbone, *R*_bb_/*R*_bb,0_ (thin red lines), as a function of the average DP of the spacer segment, *N*_g_. Regimes I and II (*N*_g_ > *N*_g_^*^, see eq 6): both the side chains and the backbone polymer adopt unperturbed Gaussian conformation. Regime III (*N*_g_^*^ > *N*_g_ > *N*_g_^**^, see eq 9): the side chain remains unperturbed, but the backbone becomes extended to mitigate the crowding among the overlapped side chains. Regime IV (*N*_g_^**^ > *N*_g_ > 1): the spacer segment between two neighboring grafting sites is fully extended. To avoid crowding, the side chain must extend radially away from the backbone.

In brief, at relatively low grafting density (*N*_g_ > *N*_g_^*^) (eq 6), there is no crowding issue among the side chains from the same grafted polymer. Thus, the side chains are unperturbed and adopt Gaussian conformation, and so does the backbone polymer (Regimes I and II in Figure 2). As the grafting density increases, the volume between two neighboring grafting sites is insufficient to accommodate a side chain. Yet, the backbone polymer can extend to avoid the crowding of side chains. This is reminiscent of pulling the two ends of the backbone to increase its end-to-end distance. As the grafting density increases, the side chain conformation remains Gaussian-like. By contrast, the backbone continues to extend to ensure a constant distance in space between two neighboring grafting sites of side chains, such that the side chains are not crowded (Regime III in Figure 2). However, the extension of the backbone cannot continue forever; instead, it will stop at a certain grafting density *N*_g_^**^, at which the section of the backbone polymer between two neighboring grafting sites is stretched to its contour length (eq 9). At high grafting density with 1 < *N*_g_ < *N*_g_^**^, there is no other way for the side chains to avoid crowding except by extending radially away from the backbone polymer. The end-to-end distance of the side chain increases at higher grafting density (Regime IV in Figure 2). Below, we summarize the theory for the molecular structure of cBB polymers in each of these four regimes.

### 3.1 Loose Comb: Low Grafting Density (*N*_g_ > *N*_sc_)

In Regime I with low grafting density of *N*_g_ > *N*_sc_, two neighboring side chains from the same grafted polymer do not overlap with each other. The grafted polymer is reminiscent of a loose comb, and both the side chains and the backbone adopt unperturbed Gaussian conformation. The root-mean-square end-to-end distance of a side chain is the random walk of Kuhn segments with length *b*_sc_
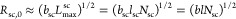
1Here, *L*_max_^sc^ is the contour length of the side chain
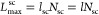
2

Similarly, the size of the unperturbed backbone polymer is
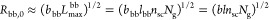
3in which *n*_sc_*N*_g_ is the DP of the backbone polymer, and *L*_max_^bb^ is the contour length of the backbone polymer.
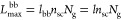
4

### 3.2 Dense Comb: Intermediate Grafting Density (*N*_g_^*^ < *N*_g_ < *N*_sc_)

As the grafting density becomes higher with *N*_g_ < *N*_sc_, the side chains from the same grafted polymer start to overlap. Thus, the grafted polymer is called a “dense comb”. The conformations for both the side chains and the backbone, however, remain unperturbed until a crossover grafting density *N*_g_^*^, at which the side chains from the same grafted polymer are enough to completely fill the volume pervaded by one side chain, *V*_P_ ≈ *R*_sc,0_^3^
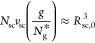
5Here, *g* is the number of monomers of a section of the backbone polymer passing through the pervaded volume, and *g*/*N*_g_^*^ corresponds to the number of side chains within *V*_P_. Because the size of the backbone section is about that of the side chain, , eq 5 can be rewritten as
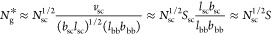
6Here *S*_sc_ is a dimensionless parameter that is determined by the aspect ratio of the side chain Kuhn segment
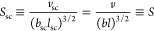
7

For typical polymers, the value of *S* is less than 1. For example, for a linear PDMS with *b* = 13.0 Å, *l* = 2.55 Å, and *v* = 127 Å^3^, *S* = 0.7; whereas for a linear poly(benzyl methacrylate) (PBnMA) with *b* = 18.6 Å, *l* = 2.56 Å, and *v* = 248 Å^3^, *S* = 0.8 (Table 1).

**Table 1:** Polymer Physics Parameter of Different Polymers^a^

	*C*_*∞*_	cos(θ/2)	*l*_0_ (Å)	*m*_0_ (g/mol)	*M*_0_ (g/mol)	ρ (g/cm^3^)	*N*_K_	*b* (Å)	*l* (Å)	*v* (Å^3^)	*v*_K_ (Å^3^)
PDMS	5.8^b^	NA^c^	1.64	74	381^d^	0.965	5.1^e^	13.0^d^	2.55^e^	127	650
PBnMA	10.0^f^	0.83	1.54	176	1276	1.18	7.3	18.6	2.56	248	1796
PMMA	9.0	0.83	1.54	100	655	1.18	6.6	17.0	2.56	141	923

a *C*_*∞*_, Flory’s characteristic ratio; *l*_0_, length of one main-chain bond; *m*_0_, mass of a chemical monomer; *M*_0_, mass of a Kuhn segment; ρ, polymer density; *N*_K_, number of chemical monomers per Kuhn segment; *b*, length of a Kuhn segment; *l*, length of a chemical monomer; *v*, volume of a chemical monomer; *v*_K_, volume of a Kuhn segment. The length *b* of a Kuhn segment for a linear polymer is , in which θ is the bond angle. For PDMS, each chemical monomer has two Si–O bonds; similarly, for methacrylate-based polymer, each chemical monomer has two C–C bonds.

b Flory ratio for linear PDMS with the number of repeating units around 15; for extremely long chains, *C*_*∞*_ approches to 6.43.^53^

c For PDMS, there are two bond angles: 110° for ∠OSiO and 143° for ∠OSiO; thus, the correlation  is not applicable to PDMS.

d Data from ref (54).

e Values back calculated based on existing parameter: *N*_K_ = *M*_0_/*m*_0_, and *l* = *b*/*N*_K_.

f Data from ref (55).

### 3.3 Loose Bottlebrush: Intermediate High Grafting Density (*N*_g_^**^ < *N*_g_ < *N*_g_^*^)

As the grafting density further increases with *N*_g_ < *N*_g_^*^, the volume pervaded by a side chain is not enough to accommodate all side chains from the same grafted polymer passing through the pervade volume if the backbone conformation remains unperturbed. To avoid the crowding of side chains, the backbone polymer must be extended to increase the distance between two neighboring grafting points to *R*_sc,0_/*P*_sc_. Here, *P*_sc_ is the number of side chains within the pervade volume, *R*_sc,0_^3^, of one side chain
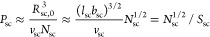
8

This ensures that the number of side chains is just enough to completely fill the pervade volume of one side chain. During this process, the conformation of side chains remains unperturbed. Yet, the backbone polymer becomes more extended as the grafting density increases or *N*_g_ decreases. This trend cannot continue forever, however, as the extension of the backbone must stop at a certain grafting density, *N*_g_^**^, at which the backbone section between two neighboring grafting sites is stretched to its maximum length, *l*_bb_*N*_g_^**^ ≈ *R*_sc,0_/*P*_sc_. Recall eqs 1 and 8, one obtains
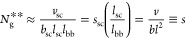
9Here *s*_sc_ is a packing parameter associated with the ratio of the volume of a chemical monomer to the volume of a rod-like Kuhn segment.
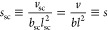
10

For *N*_g_^**^ < *N*_g_ < *N*_g_^*^, the conformation of the backbone polymer can be visualized as a series of tension blobs of size ξ_t_ up to the length scale of *R*_sc,0_.
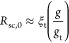
11where *g*_t_ is the number of monomers per tension blob, and *g* is the number of monomers per backbone polymer section of size *R*_sc,0_.

At length scales smaller than the tension blob, the backbone section does not feel crowding and adopts unperturbed Gaussian conformation: . There are *g*_t_/*N*_g_ side chains grafted to the backbone within length scale ξ_t_. Thus, the total number of monomers from all side chains with a section of size ξ_t_ is *g*_sc_*g*_t_/*N*_g_, where *g*_sc_ is the number of side chain monomers with a section of size ξ_t_ and is given by relation: . The crowding would occur if the total volume of all the side chain sections reaches the volume of the tension blob: ξ_t_^3^ ≈ *v*_sc_*g*_sc_*g*_t_/*N*_g_. Using eq 9, this condition gives the number of monomers per tension blob
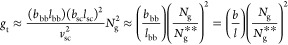
12

The size of the tension blob is
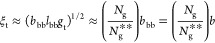
13

Note that at *N*_g_^**^ (eq 9), the size of the tension blob is about the Kuhn length of the backbone polymer ξ_t_ ≈ *b*_bb_, which is the smallest length scale at which the scaling theory applies.

At length scales larger than *R*_sc,0_, the backbone polymer is a random walk of blobs with size of *R*_sc,0_:
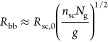
14

Using eqs 1 and 11–13, the number of monomers per blob, *g*, can be obtained
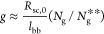
15

Recall the expression for *R*_bb,0_ (eq 3), one obtains
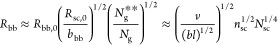
16

In Regime III, the size of the backbone increases with the grafting density by a power of 1/2, as shown by the thin red line in Figure 2. Moreover, the side chains from the same grafted polymer are sufficient to fill the space near the backbone of the grafted polymer. Yet, the side chains adopt unperturbed Gaussian conformation. Thus, the grafted polymer is termed a “loose bottlebrush”.

### 3.4 Dense Bottlebrush: High Grafting Density (1 < *N*_g_ < *N*_g_^**^)

In this regime, the section of the backbone polymer between two neighboring grafting sites is already fully stretched. To avoid crowding, the side chains must be stretched radially away from the bottlebrush backbone. This results in a filament-like bottlebrush polymer with a diameter about the size of the side chain. The side chain size *R*_sc_ is determined by volume conservation: *R*_sc_^2^*N*_g_*l*_bb_ ≈ *v*_sc_*N*_sc_. Using eqs 1 and 9, one obtains
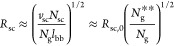
17Here and below, we ignore the difference in polymer density as for most polymers it is nearly the same of ∼1 g/cm^3^. Equation 17 suggests that at high grafting density, *N*_g_ < *N*_g_^**^, the conformation of a side chain remains ideal, but with its size increasing with the grafting density by a power of 1/2 (Regime IV, Figure 2). At the highest grafting density with one side chain per backbone chemical monomer (*N*_g_ = 1), the side chain is stretched by a factor of .

In Regime IV, the whole grafted polymer becomes a densely grafted bottlebrush, which can be considered as a ‘fat’ linear polymer with a persistence length, , about the cross-section of the bottlebrush: . At length scales smaller than the persistence length, the backbone polymer section is nearly fully extended with the number of backbone monomers of *R*_sc_/(*N*_g_*l*_bb_). Thus, the size of the bottlebrush is . Recall eqs 3 and 17, one obtains
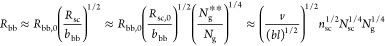
18

This suggests that the size of the bottlebrush backbone increases with the grafting density by a power of 1/4 (Regime IV, Figure 2).

One can estimate the values of *N*_g_^*^ and *N*_g_^**^ for a bottlebrush polymer consisting of methacrylate-based backbone and PDMS side chains by assuming that the backbone and side chains are compatible (χ = 0). We consider PDMS side chains of MW *M*_sc_ = 5000 g/mol, which is relatively large but accessible to typical polymer synthesis methods;^15^ this MW corresponds to *N*_sc_ = *M*_sc_/*m*_0_ ≈ 68 chemical monomers, where *m*_0_ is the mass of a PDMS chemical monomer (see Table 1). The polymer physics parameters of PDMS and two kinds of methacrylate-based backbone polymers are listed in Table 1. The value of *N*_g_^**^ (eq 9) for PDMS bottlebrush polymers with a methacrylate-based backbone is
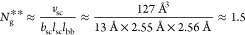
19

The value of *N*_g_^*^ (eq 6) depends on the Kuhn length of backbone polymer. For a poly(benzyl methacrylate) (PBnMA) backbone
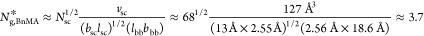
20

This value becomes slightly larger for poly(methyl methacrylate) (PMMA) backbone
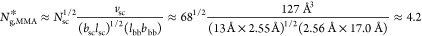
21

These results indicate that for a typical bottlebrush polymer the window for the densely grafted bottlebrush (1<*N*_g_ < *N*_g_^**^) is relatively narrower compared to that for the loosely grafted bottlebrush (*N*_g_^**^<*N*_g_ < *N*_g_^*^). Moreover, the value of *N*_g_^*^ is smaller than the number of chemical monomers per Kuhn segment, *N*_K_ ≈ 7 (Table 1). This suggests that the grafting density must be relatively high (low *N*_g_) to ensure the grafted polymer is bottlebrush-like.

Based on the value of the crossover grafting density *N*_g_^**^ ≈ 1.5, one can estimate the entropic free energy penalty associated with stretching the bottlebrush backbone and side chains. At the highest grafting density (*N*_g_ = 1), a side chain is stretched by  times (eq 17), and the backbone is stretched by  times (eq 18), in which  for PMMA backbone. For a bottlebrush polymer with 200 side chains (*n*_sc_ = 200), the ratio between the entropic free energy associated with stretching the side chains, *F*_sc_, and that associated with stretching the backbone, *F*_bb_, is . Thus, for a densely grafted bottlebrush with many side chains (*n*_sc_ ≫ *N*_sc_), the entropic free energy penalty due to chain extension is dominated by the contribution from stretched side chains but not the bottlebrush backbone. That is also why the backbone, not the side chains, is stretched first in Regime III.

## 4 Foldable Bottlebrush Polymers with Incompatible Backbone and Side Chains

We extend the seminal work by Rubinstein Lab^50^ to a general scenario, in which a grafted polymer consists of incompatible backbone and side chains. The incompatibility between the two distinct polymer species is described by the Flory–Huggins interaction parameter χ. We restrict our consideration to cases with highly incompatible backbone and side chains (e.g., χ ∼ 0.1 or higher) such that the interface between domains formed by different polymer species is sharp.^56,57^

To reduce interfacial free energy, the backbone polymer is prone to phase separate from the side chains. However, unlike a binary mixture consisting of two immiscible molecules such as water and oil, or a polymer blend consisting of incompatible polymers such as polystyrene and PDMS, where the minority phase tends to form spherical droplets to minimize interfacial area, the microphase separation within the grafted polymer is constrained by chain connectivity. As the backbone polymer folds, it pulls the side chains closer. This process effectively decreases the distance between two neighboring grafting sites in space, which may cause the crowding of side chains. If that occurs, the side chains must extend, resulting in increased entropic free energy associated with stretching the side chains.

Additionally, the spacer segment must span the smallest dimension of the domain collapsed by the backbone polymer. This condition ensures that all grafting sites are located at the surface of the backbone domain, such that there is no mixing between side chains and the backbone polymer. This is the major difference between fBB and cBB polymers, where the incompatibility between the side chains and backbone polymer is ignored, such that they can be mixed without enthalpic penalty. At relatively high grafting density (small spacer segment DP), the spacer segment may not be able to main unperturbed Gaussian conformation; instead, it must be stretched to span the smallest dimension of the backbone domain. This process results in an entropic penalty associated with stretching the backbone polymer. Consequently, the equilibrium molecular structure of the grafted polymer is determined by the balance between interfacial free energy and entropic penalty attributed to chain stretching
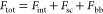
22where *F*_tot_ is the total free energy of a grafted polymer, *F*_int_ is the interfacial free energy between the incompatible backbone and side chains, *F*_sc_ and *F*_bb_ are, respectively, the entropic free energies due to the stretching of the side chains and the backbone polymer.

**Figure 3:**
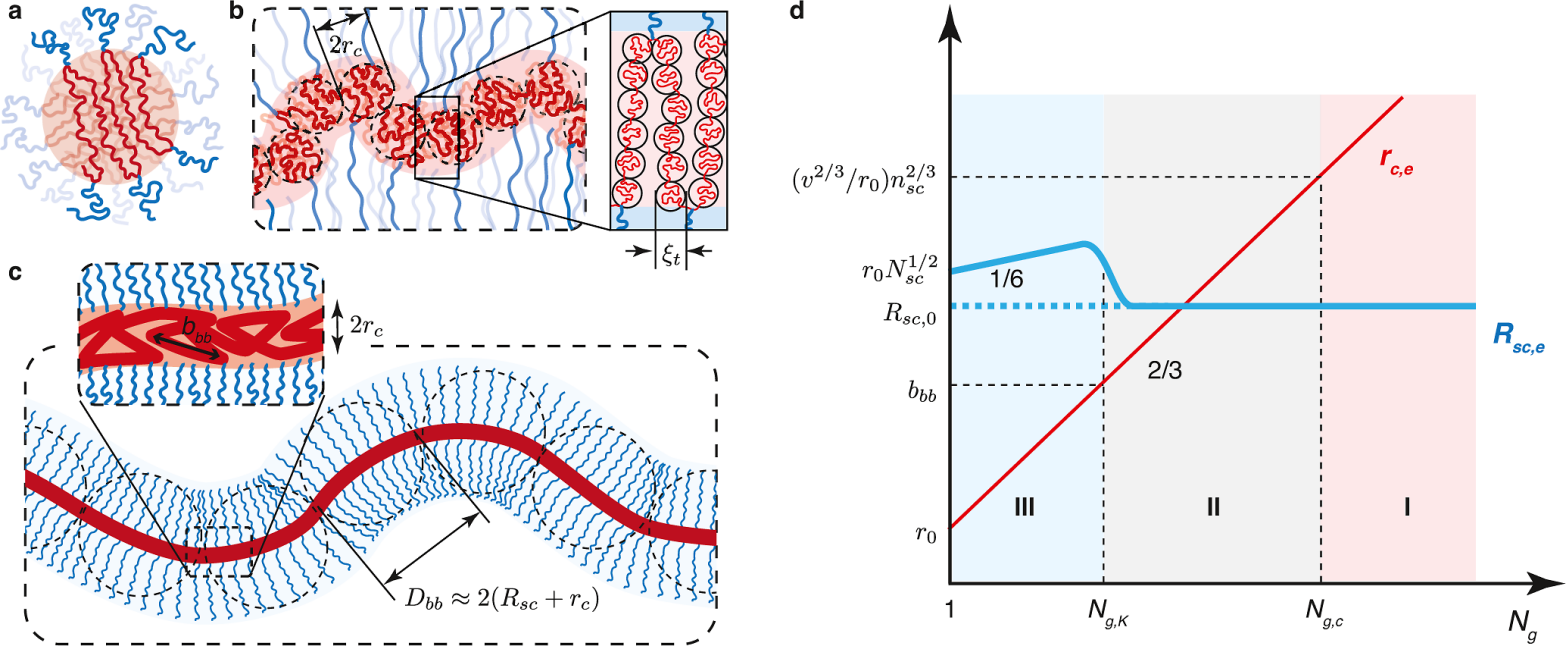
Molecular structure of a foldable bottlebrush polymer in the melt. (a) At low grafting density with *N*_g_ > *N*_g,c_ (eq 32), the backbones of multiple grafted polymers aggregate to a spherical core with its surface grafted by side chains. Within the spherical core, the spacer segments are stretched to balance interfacial tension. (b) At intermediately grafting density with *N*_g,c_ > *N*_g_ > *N*_g,K_ (eq 41), the backbone polymer collapses into a cylindrical core with an equilibrium cross-section size *r*_c,e_. This size is determined by the spacer segment, which is a stretched array of tension blobs. (c) At high grafting density with *N*_g,K_ > *N*_g_ > 1, the backbone still collapses into a cylindrical core but with the cross-section size smaller than the Kuhn length of a backbone polymer, *b*_bb_, such that the backbone Kuhn segments are packed along the contour of the cylindrical core. The cross-section size of the core is determined by the balance between the interfacial free energy of distinct domains and the entropic free energy penalty attributed to stretching side chains and confining the backbone into a slim cylinder. (d) Scaling regimes for the dependence of equilibrium sizes of the side chain, *R*_sc,e_, and the radius of the collapsed backbone, *r*_c,e_, on the average DP of the spacer segment *N*_g_. Regime I (*N*_g_ > *N*_g,c_, eq 32): the backbone collapses into a sphere with *r*_c,e_∝*N*_g_^2/3^ (eq 31) and the side chain adopts unperturbed Gaussian conformation, as illustrated in panel (a). At *N*_g_ ≈ *N*_g,c_, *r*_c,e_ ∝ *n*_sc_^2/3^ (eq 31). Regime II (*N*_g,c_ > *N*_g_ > *N*_g,K_, eq 41): the backbone collapses into a thick cylinder with *r*_c,e_ ∝ *N*_g_^2/3^ (eq 38), and the side chain adopts unperturbed Gaussian conformation. In the cylindrical core, the backbone folds back and forth with the spacer segment being stretched to span the cross-section of the cylinder, as illustrated in panel (b). Regime III (*N*_g,K_ > *N*_g_ > 1): the backbone collapses into a slim cylinder with a radius of *r*_c,e_∝*N*_g_^2/3^ (eq 55), and the side chains are stretched radially away from the backbone to avoid crowding with size of *R*_sc,e_ ∝ *N*_sc_^1/2^*N*_g_^1/6^ (see eq 56). The crossover profile of the side chain size between Regimes III and II remains an open question subject to future explorations. In Regime III, a key feature is that the size of side chain increases with the decrease of the grafting density. For foldable bottlebrush polymers, the radius of the backbone domain (*r*_0_) at the highest grafting density (*N*_g_ = 1) is determined by the interfacial tension γ, or Flory–Huggins interaction parameter χ, between the side chain and bottlebrush backbone polymer (eq 25).

We identify *three* regimes depending on the grafting density of side chains (1/*N*_g_). At low grafting density (*N*_g_ > *N*_g,c_) (eq 32), the backbones of multiple grafted polymers aggregate to form a spherical core with the spacer segment being stretched to compensate for interfacial tension (Figure 3a). At the crossover grafting density (*N*_g_ ≈ *N*_g,c_), there is only one grafted polymer within the spherical structure. At intermediate grafting density with *N*_g_ larger than the DP of a Kuhn segment (*N*_g,c_ > *N*_g_ > *N*_g,K_) (eq 41), the backbone collapses to a thick cylinder with the spacer segment being stretched to cross the cylinder cross-section (Figure 3b). Yet, the side chains are not crowded and adopt unperturbed Gaussian conformation. At high grafting density (*N*_g,K_ > *N*_g_ > 1), the backbone polymer collapses to a slim cylinder with its surface densely grafted with many side chains that are radially stretched away from the backbone (Figure 3c). Below, we describe in detail the scaling theory and physical pictures for each regime.

### 4.1 Sphere: Low Grafting Density (*N*_g_ > *N*_g,c_)

At low grafting density, multiple grafted polymers aggregate to a micelle structure with either side chains or backbones as the core. To form a micelle with side chains as the core, it requires *N*_sc_ > *n*_sc_ (see Supporting Information). However, in this paper, we restrict our consideration to *n*_sc_ ≫ *N*_sc_. Thus, we focus on the micelle structure in which the backbone aggregates to a spherical core with grafting sites located at the sphere surface, as illustrated in Figure 3a. This configuration minimizes the interfacial free energy between the side chain domain and the backbone domain.

The radius of the spherical domain, *r*_c_, is determined by the mass conservation of the backbone polymer
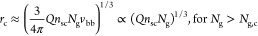
23Here, the aggregation number *Q* corresponds to the number of grafted polymers per micelle.

The interfacial free energy of the spherical domain is proportional to the surface area of the sphere
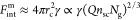
24Here, γ is the polymer–polymer interfacial tension for an asymmetric polymer binary mixture, which is correlated to the Flory–Huggins interaction parameter, χ^56,57^
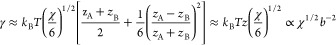
25where *k*_B_ is Boltzmann constant, *T* is the absolute temperature, and *z*_*i*_ = *b*_*i*_/*v*_K,*i*_. Here, *b*_*i*_ and *v*_K,*i*_, respectively, are the Kuhn segment and specific volume of the Kuhn monomer for species *i* being polymer A and B.

Reminiscent of micelles self-assembled by classical asymmetric AB diblock copolymers,^58^ within the spherical domain, each spacer segment is stretched from its unperturbed size, *r*_g,0_, to the size of the spherical domain, *r*_c_, to ensure that all grafting sites are at the surface of the spherical domain.
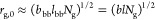
26

This process results in an entropic penalty associated with stretching a spacer segment from *r*_g,0_ to *r*_c_: *k*_B_*Tr*_c_^2^/*r*_g,0_^2^. Since there are *n*_sc_ spacer segments per grafted polymer, the entropic free energy attributed to stretching the backbone of one grafted polymer is
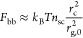
27

The total free energy of an individual grafted polymer within the micelle is
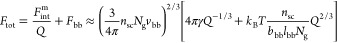
28

Minimizing the free energy gives the equilibrium aggregation number Q^*^
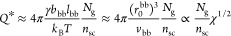
29

Here, *r*_0_^bb^ is a length scale determined by the Flory–Huggins interaction parameter χ and the polymer physics parameters of the backbone polymer
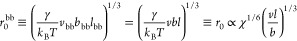
30

Substituting eq 29 into eq 23 obtains the equilibrium size of the spherical core
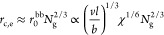
31

Equation 31 suggests that size of the spherical core increases with the spacer segment DP by a power of 2/3.

This multiple-polymer aggregation ends at a crossover grafting density *N*_g,c_, at which there is only grafted polymer per micelle (Q^*^ = 1). Recall eq 29, one obtains
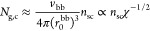
32

Expression eq 32 suggests that the crossover value *N*_g,c_ is not affected by the molecular weight of the side chain. Instead, it is proportional to the number of side chains per grafted polymer  as the spherical domain must be large enough to ensure all grafting sites on its surface. Moreover, for polymers with higher extent of incompatibility (larger γ or χ), the backbone domain can remain spherical until a higher crossover grafting density (1/*N*_g,c_). This is because the loss in interfacial free energy and can compensate for a higher extent of chain stretching. In Regime I, the side chains adopt unperturbed Gaussian conformation with *R*_sc_ = *R*_sc,0_ as they are far apart from each other (see eq 1).

One can estimate the length scale of *r*_0_^bb^ (eq 30) for highly incompatible side chains and backbone polymer. For instance, for a grafted polymer with PMMA backbone and PDMS side chains, the interfacial tension γ ≈ 10^–2^ N·m^–1^, *b*_bb_ ≈ 1.7 nm, *l*_bb_ ≈ 2.56 Å, and *v*_bb_ ≈ 140 Å^3^. Substituting these values into eq 30, one obtains *r*_0_^bb^ ≈ 0.5 nm, which is less than 1/3 of the Kuhn segment size. Additionally, the value of  is approximately on the order of unity. Thus, the value of *N*_g,c_ is about 1 order of magnitude lower than *n*_sc_ (eq 32).

### 4.2 Thick Cylinder: Intermediate Grafting Density (*N*_g,K_ < *N*_g_ < *N*_g,c_)

As the spacer segment becomes smaller (*N*_g_ < *N*_g,c_), the backbone polymer cannot collapse to a spherical domain while keeping all grafting sites at the surface of the sphere. Instead of forming a sphere, the backbone polymer forms a cylinder with a diameter small enough for the spacer segment to span over. This process ensures that all grafting sites are located at the surface of the backbone domain, as illustrated by Figure 3b. This structure is reminiscent of the necklace configuration observed in solutions of hydrophobic polyelectrolytes, which is characterized by polymeric globules (spheres) connected by extended sections of polymer chain (strings).^59,60^ This phenomenon arises because the correlation-induced attraction of condensed counterions to charged monomers can be balanced by long-range electrostatic repulsion between uncompensated charges. By contrast, in the melt of fBB polymers, there is no long-range repulsion. Furthermore, since the backbone is grafted with side chains, forming extended backbone sections between neighboring spheres is energetically unfavorable. As a result, neighboring spheres come into direct contact, effectively forming a cylindrical core.

The interfacial free energy between the side chains and the backbone polymer is
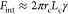
33The contour length *L*_c_ of the cylinder is determined by the volume conservation of the backbone polymer
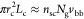
34Thus, one can rewrite eq 33 as
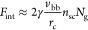
35This expression suggests that for a fixed grafting density, the thicker the backbone domain, the lower the interfacial free energy. However, increasing the diameter of the cylinder would result in stronger stretching of the spacer segment. The entropic free energy attributed to stretching the spacer segments of the backbone polymer is (eq 27)
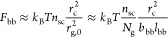
36The total free energy of the grafted polymer is
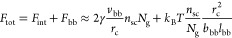
37Recalling the expression of *r*_0_^bb^ (see eq 30) and minimizing the total free energy give the equilibrium cross-section size of the cylindrical core
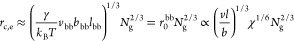
38This expression suggests that the diameter of the cylinder increases with the spacer segment by a power 2/3 (Regime II, thin red line in Figure 3d). This scaling relation is the same as that in the micelle (Regime I) (eq 31). At equilibrium, the free energy of the grafted polymer is obtained by substituting eq 38 into eq 37
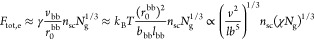
39

In Regime II, the spacer segment is a stretched array of tension blobs to span the cross-section of the cylinder: *r*_c,e_≈(*N*_g_/*g*_t_) ξ_t_, in which *g*_t_ is the DP of the polymer section within the tension blob, as illustrated in Figure 3b. Unlike conventional bottlebrush polymers in which a tension blob of the stretched backbone is filled with side chains, in a fBB polymer the tension blob is filled with spacer segments from the bottlebrush backbone. To avoid mixing the side chains with the backbone, multiple spacer segments fold back and forth to ensure that the grafting sites are located at the cylinder surface, as illustrated by the inset of Figure 3b. At length scales smaller than ξ_t_, the backbone adopts an unperturbed Gaussian conformation: . Thus, recall the expression of *r*_c,e_ (eq 38), the size of the tension blob is
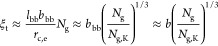
40Here, *N*_g,K_ corresponds to the grafting density at which the tension blob size is about the backbone Kuhn segment length *b*_bb_.
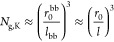
41

At *N*_g_ = *N*_g,K_, the spacer segment is nearly fully stretched to its contour length
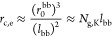
42

Expression eq 41 suggests that the lower limit of Regime II is determined by the ratio between *r*_0_^bb^ and the backbone chemical monomer length *l*_bb_. For a grafted polymer with a flexible PMMA backbone and PDMS side chains, *l*_bb_ ≈ 2.56 Å and *r*_0_^bb^ ≈ 0.5 nm (see eq 30). This gives the value of *N*_g,K_ ≈ 7.5, which is comparable to the number of chemical monomers per PMMA Kuhn segment, *N*_K,bb_ ≈ 7 (Table 1).

Alternatively, the lower limit of Regime II (*N*_g_ ≈ *N*_g,K_ ≈ *N*_K,bb_) can be understood in the context of side chain packing. Let us consider a section of the grafted polymer with length *b*_bb_ along the contour of the cylinder of cross-section size *b*_bb_. At *N*_g_ ≈ *N*_K,bb_, the number of side chains grafted to this section is *b*_bb_^3^/(*N*_g_*v*_bb_) = *b*_bb_^3^/(*N*_K,bb_*v*_bb_) ≈ *b*_bb_^2^*l*_bb_/*v*_bb_, in which *b*_bb_ ≈ *N*_K,bb_*l*_bb_. Thus, the volume of side chains grafted to the section of the cylindrical core is *V*_sc_ = *N*_sc_*v*_sc_(*b*_bb_^2^*l*_bb_/*v*_bb_). If the side chains were not stretched, the maximum volume available to the side chains near the cylindrical core is *V*_av_ = *R*_sc,0_^2^*b*_bb_. Thus, one can define a crowding parameter *p* as the ratio of the volume of side chains from the same grafted polymer, *V*_sc_, to the available volume, *V*_av_: *p* = *V*_sc_/*V*_av_. For *p* < 1, the volume of side chains from the same grafted polymer is not enough to completely fill the volume pervaded by one side chain. As a result, the side chains are not stretched and adopt unperturbed Gaussian conformation. For *p* > 1, the side chains will experience crowding if they maintain an unperturbed size *R*_sc,0_.

At grafting density with *N*_g_ ≈ *N*_K,bb_, the value of *p* is
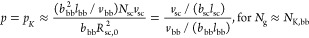
43

Ignoring the difference in polymer physics parameters between the backbone and the side chains, *p*_K_ ≈ 1. This suggests that at the grafting density of one side chain per Kuhn segment (*N*_g_ ≈ *N*_K,bb_), the available space near the backbone is just enough to accommodate all the side chains from the same grafted polymer. Thus, in Regime II, the side chains do not experience crowding and always adopt unperturbed Gaussian conformation with a constant mean-square end-to-end distance of *R*_sc,0_ (see eq 1).

Note that scaling theory does not account for bending-induced stretching of side chains. To maintain uniform density in the melt, bottlebrush polymers inevitably bend. This bending reduces the space available to the side chains on the concave side, causing them to become more stretched. By contrast, the opposite behavior occurs for the side chains on the convex side. Such variations in the stretching of side chains due to bottlebrush bending originate from thermal fluctuation. Thus, on average, the entropic penalty associated with bending-induced stretching of side chains is negligible. However, one potential phenomenon associated with bottlebrush bending is the interpenetration among side chains from neighboring bottlebrush polymers. Qualitatively, it is widely accepted that the extent of interpenetration increases at lower grafting densities. Yet, a quantitative understanding of the dependence of the depth of interpenetration on grafting density is beyond the scope of this paper and will be the subject of future explorations.

Within a grafted polymer, the backbone collapses when χ is high (the side chains and the backbone are highly incompatible). By contrast, when χ approaches zero (χ → 0), the backbone can mix homogeneously with the side chains. The onset of microphase separation within a grafted polymer can be estimated by comparing the free energy of the thick cylinder configuration with that of the disordered state. For simplicity, we ignore the differences in polymer physics parameters between the backbone and the side chains. Recalling the total free energy of a thick cylinder, *F*_tot,e_ (eq 39), the free energy of single spacer and one side chain, *F*_sp_^cyl^, is
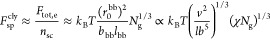
44

The free energy of a single spacer and one side chain in the disordered state is
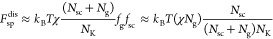
45

Here, *N*_K_ is the DP of a Kuhn segment; *f*_g_ ≈ *N*_g_/(*N*_g_ + *N*_sc_) and *f*_sc_ = 1 – *f*_g_ are, respectively, the volume fractions of the spacer segment. Equating *F*_sp_^cyl^ and *F*_sp_^dis^ gives the “order-disorder-transition” (ODT) value of χ*N*_g_
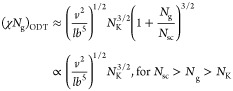
46

Similar to classical block copolymer self-assembly,^61^ χ*N*_g_ can be termed the “segregation strength” within a grafted polymer. The value of  delineates the boundary between foldable and conventional bottlebrush configurations: (i) above , the backbone collapses into a domain distinct from the side-chain domain; (ii) below , the backbone mixes homogeneously with the side chains. Note that eq 46 only provides a scaling relation for , and its exact value has yet to be determined by analytic calculations, such as self-consistent field theory.^62^ Additionally, the above argument for the onset of microphase separation applies only to relatively large spacer segments (*N*_g_ > *N*_K_). Below the Kuhn length (*N*_g_ < *N*_K_)—the elementary length scale of polymer physics models—the form of the Flory–Huggins mixing free energy (eq 45) no longer applies.

### 4.3 Thin Cylinder: High Grafting Density (1 < *N*_g_ < *N*_g,K_)

At high grafting density (*N*_g_ < *N*_g,K_), the backbone of a grafted polymer is completely shielded by the side chains from other grafted polymers. Within an individual grafted polymer, the backbone polymer can still fold to a cylindrical core with all grafting sites on its surface. Yet, because *N*_g_ < *N*_g,K_ ≈ *N*_K,bb_ the diameter of the cylinder must be smaller than the Kuhn length *b*_bb_ of the backbone polymer. Since the Kuhn segment is not spherical but cylindrical with an aspect ratio larger than one, the only way that the backbone polymer can be packed in such a slim cylinder is by stacking Kuhn segments along the contour of the cylinder, as illustrated in Figure 3c. This phenomenon is reminiscent of filling a long tube with a chain of cylindrical particles connected by flexible joints, and the length of each cylindrical particle is larger than the tube diameter. In doing so, all grafting sites are located at the surface rather than the interior of the cylinder, such that there is no mixing between the bottlebrush backbone and the side chains.

In this regime, the steric repulsion among the strongly overlapped side chains tends to elongate the cylindrical core, whereas the backbone polymer tends to collapse into a cylinder of a larger diameter, such that the interfacial area between the side chains and the backbone polymer can be reduced. Additionally, confining the backbone polymer into a slim cylinder with a diameter less than the polymer Kuhn segment size results in entropic penalty. Thus, the microstructure of bottlebrush polymer is determined by the balance between the interfacial free energy and the entropic penalty attributed to stretching the side chains and confining the backbone polymer into a slim cylinder.

To calculate the free energy associated with stretching the side chains (*F*_sc_), we consider a section of the cylindrical with the length of *b*_bb_. The number of side chains grafted to this cylindrical section is the ratio between the volume of the cylindrical section, *b*_bb_*r*_c_^2^, to the volume of a spacer segment, *N*_g_*v*_bb_: *b*_bb_*r*_c_^2^/(*N*_g_*v*_bb_). These side chains fill the space near the cylinder with the volume of ; this gives
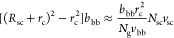
47

Assuming that *N*_sc_ ≫ *N*_g,K_, the size of the side chain is much larger than the radius of the cylinder, *R*_sc_ ≫ *r*_c_. The effects of cylindrical core on the volume available to the side chains can be neglected
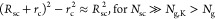
48

Therefore, one can rewrite eq 47 to obtain the size of the side chain
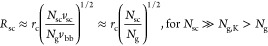
49

The entropic free energy due to the stretching of *n*_sc_ side chains of the bottlebrush polymer is
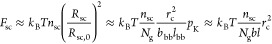
50

To calculate the free energy associated with confining the backbone polymer into a slim cylindrical tube (*F*_bb_), one can consider the backbone polymer as a freely jointed chain of rigid rods. Each rod is a Kuhn segment of length *b*_bb_. Confining a rigid rod in a small tube of diameter 2*r*_c_ < *b*_bb_ reduces the orientational degrees of freedom. In a free space, the number of degrees of freedom of a rod is on the order of . By contrast, when being confined within a cylinder, the number of degrees of freedom is on the order of . Thus, the increase in free energy per rod due to confinement is
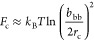
51

This result was implicitly indicated in Auvray’s work^63^ on confining an infinitely stiff rod in a tube and later was extended by Odijk in a seminal work^64,65^ to calculate the free energy of confining semiflexible polymers such as double-stranded DNA into a cylindrical pore. The confinement free energy of the whole backbone polymer with is
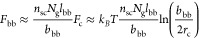
52Here, *n*_sc_*N*_g_*l*_bb_/*b*_bb_ is the number of Kuhn segments within the bottlebrush backbone.

Recall the expressions for the interfacial free energy *F*_int_ (eq 35), the entropic penalty associated with side chain stretching *F*_sc_ (eq 50), and that associated with confining the backbone polymer into a slim cylinder *F*_bb_ (eq 52), the total free energy of the collapsed bottlebrush polymer is
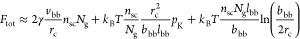
53

Since the backbone Kuhn segment length is greater than the diameter of the cylindrical core, *b*_bb_/2*r*_c_ > 1, on the right of the above equation the third term is smaller than the first one. Thus, eq 53 can be approximated as
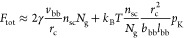
54

The approximated eq 54 originates from the fact that at high grafting density, the resistance to backbone folding is mainly from the steric repulsion among highly overlapped side chains.

Minimizing the total free energy (eq 54), one obtains the equilibrium cross-section size of the cylindrical core
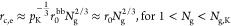
55Equation 55 suggests that in Regime III the cross-section of the cylindrical core increases with *N*_g_ by a power of 2/3, as shown by the thin red line in Figure 3d. This scaling relation is the same as that in Regime II (see eq 38). The difference is that in Regime II the side chains are not crowded and adopt unperturbed Gaussian conformation, whereas in Regime III the side chains are strongly stretched away from the collapsed bottlebrush backbone.

The equilibrium size of the side chain can be obtained by substituting eq 55 into eq 49
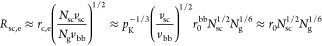
56Equation 56 suggests that the side chain size increases with the spacer size *N*_g_ by a power of 1/6: *R*_sc,e_ ∝ *N*_g_^1/6^. This behavior is qualitatively different from the case for cBB polymers (χ = 0), where the size of a side chain decreases with *N*_g_ by a power of −1/2: *R*_sc_ ∝ *N*_g_^–1/2^ (see eq 17). Such a remarkable difference originates from the strong segregation between the highly incompatible side chains and backbone polymer, which drives the backbone polymer to fold along its contour. The collapse of the backbone polymer further increases the grafting density of side chains, and therefore, results in more severe crowding of side chains, such that side chains are more extended.

Note that without spacer monomers (*N*_g_ = 1), there is no backbone folding and the side chain size is *R*_sc,e_ ≈ *r*_0_*N*_sc_^1/2^ (eq 56). The side chain size should be comparable to that in cBB polymers, *R*_sc_ ≈ (*v*/*l*)^1/2^*N*_sc_^1/2^ (eq 17). Indeed, the value of *r*_0_ is comparable to (*v*/*l*)^1/2^ for grafted polymers with highly incompatible backbone and side chains.

## 5 Comparison between Conventional and Foldable Bottlebrush Polymers

We are interested in the case where the side chains are stretched or the side chains from the same grafted polymer are sufficient to occupy the volume near the backbone, such that the grafted polymers adopt a bottlebrush-like molecular architecture. For conventional grafted polymers, this situation corresponds to relatively high grafting density (*N*_g_ < *N*_g_^*^, Regimes III and IV, Section 3.3 and Section 3.4). For fBB polymers, this situation corresponds to high grafting density (*N*_g_ < *N*_g,K_, Regime III, Section 4.3). We focus on two experimentally measurable properties: (i) the bottlebrush diameter, or the interbackbone distance *D*_bb_, and (ii) the equilibrium size of the bottlebrush polymer, *R*_bb,e_. The interbackbone distance *D*_bb_ can be directly measured by wide-angle X-ray scattering (WAXS) or neutron scattering. The equilibrium size of the bottlebrush polymer determines the maximum extent it can be stretched to
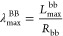
57

Additionally, to highlight how the molecular architecture affects the extensibility of a polymer, we introduce a parameter to describe the ratio between the extensibility of a bottlebrush polymer, λ_max_^BB^, and its linear counterpart, λ_max_^0^
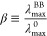
58Here,  is the extensibility of a linear polymer of the same DP (*N*_g_*n*_sc_) as the bottlebrush backbone. If β < 1, the bottlebrush polymer is prestrained and is less stretchable than its linear counterpart. If β > 1, the bottlebrush polymer is more stretchable than its linear counterpart.

### 5.1 Conventional Bottlebrush Polymers

#### 5.1.1 Interbackbone Distance

**Figure 4:**
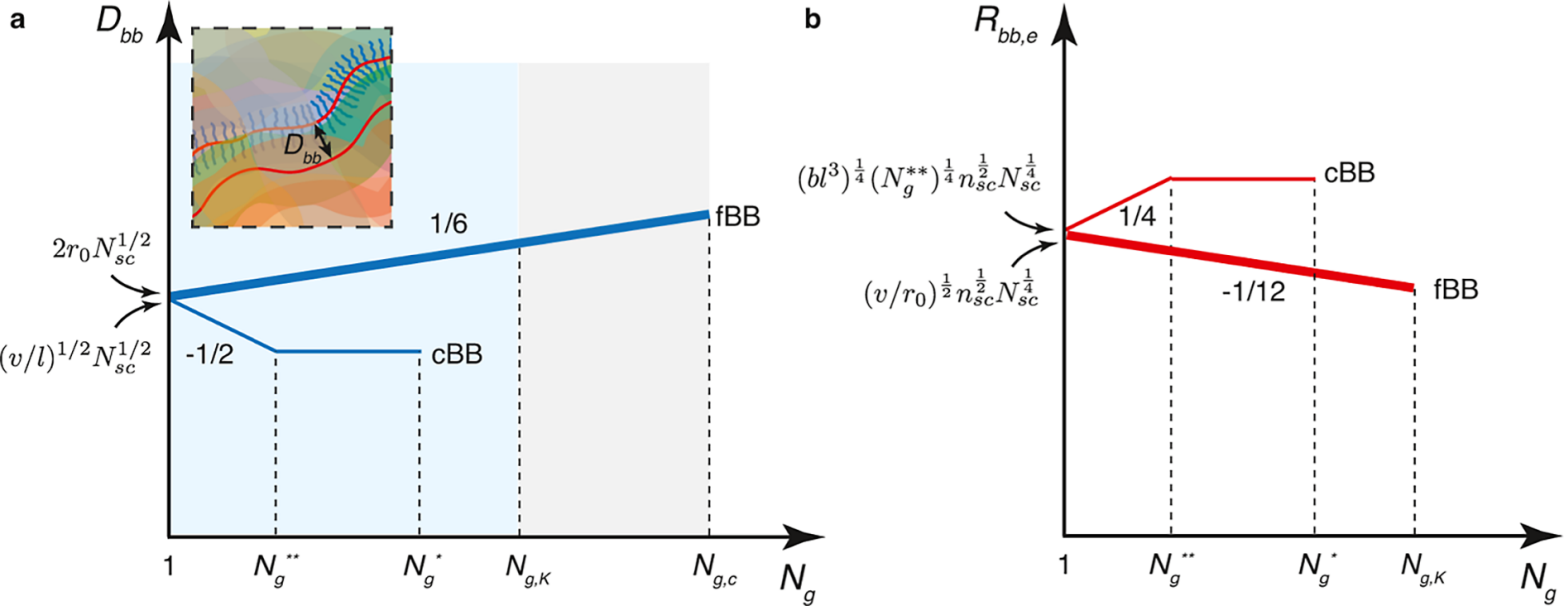
Interbackbone distance and size of bottlebrush polymers. (a) Scaling regimes for the interbackbone distance (*D*_bb_) for conventional bottlebrush (cBB) (thin line) and foldable bottlebrush (fBB) (thick line) polymers in the melt. For cBB polymers, *D*_bb_ decreases with the decrease of grafting density (increase in *N*_g_) in the dense-bottlebrush regime: *D*_bb_ ∝ *N*_sc_^1/2^*N*_g_^–1/2^ (thin line) (eq 59). In the loose bottlebrush regime, the side chains are not extended and *D*_bb_ becomes a constant. By contrast, for fBB polymers, *D*_bb_ increases monotonically with the decrease of grafting density: *D*_bb_ ∝ *N*_sc_^1/2^*N*_g_^1/6^ (thick line) (eqs 64 and 67). (b) Equilibrium end-to-end distance, *R*_bb,e_, for cBB (thin line) and fBB (thick line) polymers. Unlike cBB polymers whose size increases at higher *N*_g_ (eq 60), the size of fBB polymers decreases at higher *N*_g_:  (eq 68). This suggests that foldable bottlebrush polymers store lengths in their collapsed backbone. For cBB polymers, the crossover grafting densities, *N*_g_^*^ and *N*_g_^**^, are given by eqs 6 and 9, respectively. For fBB polymers, the crossover grafting densities, *N*_g,c_ and *N*_g,K_, are given by eqs 32 and 41, respectively. Typically, *N*_g,K_ is greater than *N*_g_^*^ (eq 21).

For a cBB polymer in the melt, the interbackbone distance equals the bottlebrush diameter, which is twice the side chain size (eq 17), as schematically illustrated in Figure 2a and shown by the thin line in Figure 4a.
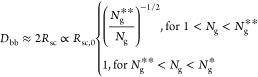
59

This suggests that the bottlebrush diameter initially decreases with the increase of spacer segment DP (*N*_g_) and then reaches the unperturbed side chain size.

#### 5.1.2 Extensibility

The size of a cBB polymer is described by eqs 16 and 18, which can be summarized as follows (thin line, Figure 4b)
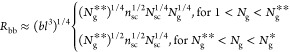
60

Compared to the unperturbed size *R*_bb,0_ of a linear polymer with the same DP as the bottlebrush backbone, the bottlebrush polymer is prestretched by an extent of
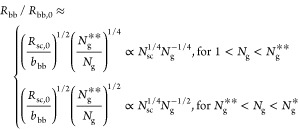
61

**Figure 5:**
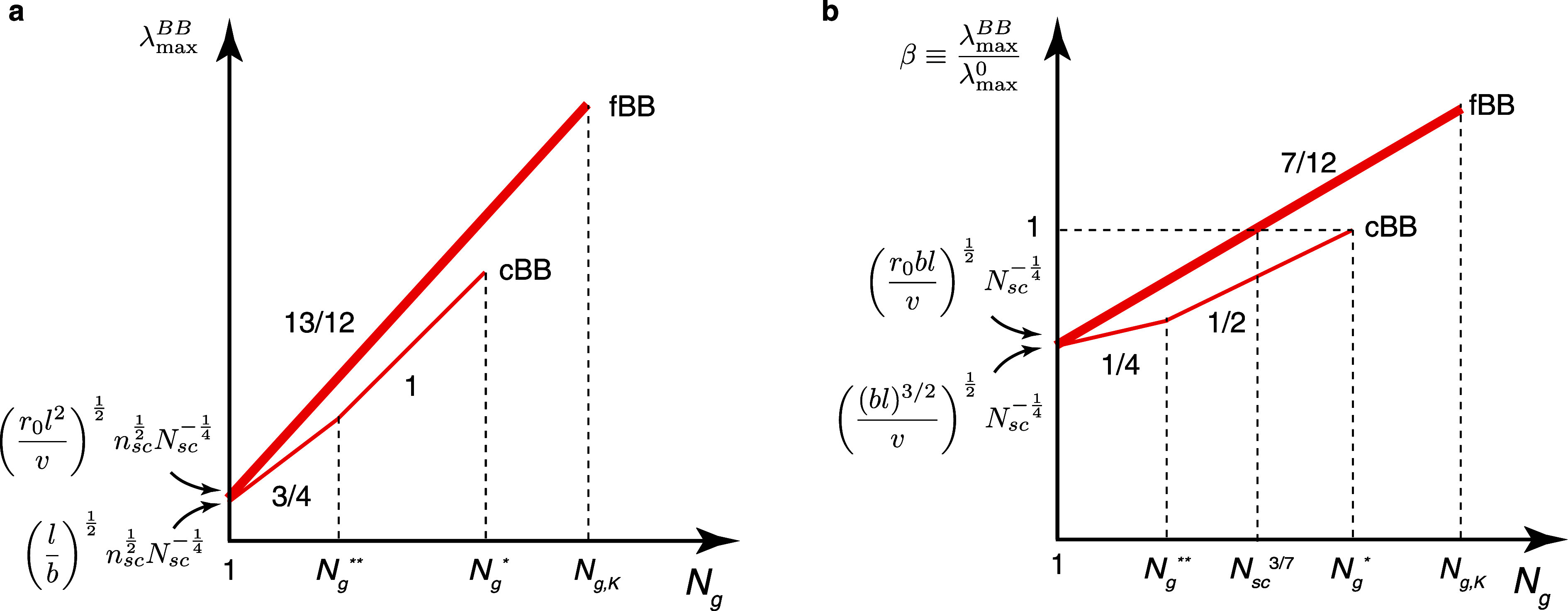
Extensibility of bottlebrush polymers. (a) Absolute extensibility λ_max_^BB^ of cBB (thin line) and fBB (thick line) polymers. The extensibility of cBB polymers (eq 62) is always lower than that of fBB polymers (eq 70). (b) The ratio between the extensi extensibility of bottlebrush polymers (λ_max_^BB^) and their linear counterpart (λ_max_^0^). For cBB polymers, it can never be more stretchable than its linear counterpart (eq 63). By contrast, fBB polymer can be more stretchable than its linear counterpart when *N*_g_ > *N*_sc_^3/7^. The value of *N*_sc_^3/7^ can be smaller than *N*_g,K_, the upper limit at which the collapsed grafted polymer remains a bottlebrush conformation (eq 71).

Since in a bottlebrush polymer, *N*_g_ < *N*_g_^*^ ≪ *N*_sc_, *R*_bb_/*R*_bb,0_ > 1; this suggests that the size of the bottlebrush is greater than its unperturbed size regardless of grafting density. Consequently, the extensibility of cBB polymers is relatively low (thin line, Figure 5a)
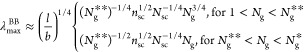
62

Indeed, compared to the extensibility of a linear polymer of the same DP, , the extensibility of cBB polymers is lower by a factor of β (thin line, Figure 5b)
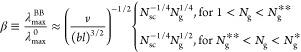
63

One can estimate the extensibility of cBB polymers with compatible backbone and side chains. An example of cBB polymers is linear PDMS side chains grafted to a long linear PDMS backbone, in which both the side chains and the backbone are linear PDMS and thus compatible (χ = 0). We use a previously reported experimental system,^13^ which has the molecular architecture parameters [*n*_sc_, *N*_sc_] of [300, 68]. Using the polymer physics parameters of linear PDMS listed in Table 1, one obtains *N*_g_^**^ ≈ 1.5 (eq 9), *N*_g_^*^ ≈ 5.3 (eq 6), and . Compared to its linear counterpart, the bottlebrush extensibility is lower by a factor β = 0.42 and 0.46 for *N*_g_ = 1 and *N*_g_^**^, respectively. Only at *N*_g_ = *N*_g_^*^, the upper limit below which the grafted polymer exhibits a bottlebrush molecular architecture, the bottlebrush extensibility matches its linear counterpart. However, the total DP of the bottlebrush backbone is very large (*N*_g_*n*_sc_ ≈ 1500), which is often difficult to be synthesized controllably. Although the extensibility of bottlebrush polymers can be increased by nearly 5 times by decreasing the side chain grafting density from the higher limit (*N*_g_ = 1) to the lower limit (*N*_g_^*^), the absolute extensibility of cBB polymers is limited (<5). Consequently, cBB polymer networks are often very brittle.

### 5.2 Foldable Bottlebrush Polymers

#### 5.2.1 Interbackbone Distance

At very high grafting density with 1 < *N*_g_ < *N*_g,K_, the bottlebrush polymer folds to a cylindrical core–shell structure, in which the shell thickness is about the size of the side chain. Thus, the interbackbone distance is about the bottlebrush diameter, which is the sum of the sizes of the side chain and the cylindrical core (eqs 55 and 56)
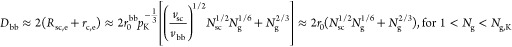
64

Expression eq 64 suggests that at high grafting density (small *N*_g_) or with long side chains (large *N*_sc_), the *D*_bb_ scales with *N*_g_ by a power of 1/6: *D*_bb_ ∼ *N*_g_^1/6^ (thick line, Figure 4a). However, the window for this regime is very small, because the diameter of the cylindrical core becomes noticeable at relatively large *N*_g_; this makes the approximation in eq 48 inappropriate. Moreover, the folding of the backbone polymer effectively increases the space available to side chains and thus alleviates the crowding of side chains. Such a correction to the side chain size is beyond the scope of this work and will be the subject of future explorations. Nevertheless, *D*_bb_ is expected to increase with *N*_g_ but with an exponent intermediate between 1/6 and 2/3.

At intermediate grafting density (*N*_g,K_ < *N*_g_ < *N*_g,c_), although the backbone still collapses into a cylindrical core, the side chains are not stretched and adopt unperturbed Gaussian conformation. Therefore, side chains from neighboring grafted polymers partially interpenetrate each other. Yet, one can estimate the interbackbone distance by considering that the side chains of the same grafted polymer fill a shell of thickness, *h*_sc_, near the cylindrical core: . Recall *L*_c,e_ = *v*_bb_*N*_g_*n*_sc_/(π*r*_c,e_^2^), one obtains
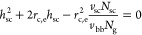
65Solving this equation, one obtains the shell thickness
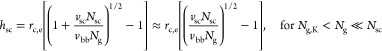
66The interbackbone distance between two neighboring grafted polymers is
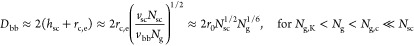
67

This expression (eq 67) suggests that even at intermediate grafting density when the side chains are not extended (Regime II), the interbackbone distance between two neighboring folded grafted polymers still increases at lower grafting density. These results suggest that for fBB polymers the interbackbone distance increases with *N*_g_ by the same power 1/6 within a wide range of side chain grafting density (thick line, Figure 4a).

#### 5.2.2 Extensibility

The conformation of the folded bottlebrush polymer can be considered as a random walk of effective Kuhn segments of size *D*_bb_: , where *L*_c,e_ is the equilibrium contour length of the cylindrical core collapsed by the backbone. It is determined by the volume conservation of the backbone polymer: *L*_c,e_ ≈ *n*_sc_*N*_g_*v*_bb_/*r*_c,e_^2^. Recall the expressions for *D*_bb_ (eq 64) and for *r*_c,e_ (eq 55), the equilibrium end-to-end distance of the bottlebrush polymer is
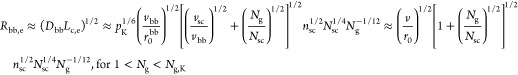
68

Since the DP of the spacer segment is much smaller than that of the side chain, *N*_g_ ≪ *N*_sc_, the above expression can be approximated as
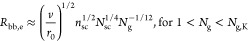
69

This suggests that size of a fBB polymer decreases at lower grafting density (*R*_bb,e_ ∝ *N*_g_^–1/12^; thick line, Figure 4b). This behavior is qualitatively different from cBB polymers, whose size increases with *N*_g_ by a power 1/4 (*R*_bb_ ∝ *N*_g_^1/4^) (eq 60). This remarkable difference has very important implications: One can use fBB polymers to store lengths in the collapsed backbone.

The extensibility of a fBB polymer is (thick line, Figure 5a)
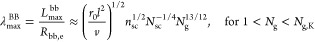
70

The ratio between the extensibility of a fBB polymer and its linear counterpart is
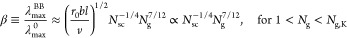
71

Equation 71 suggests that above a crossover spacer segment size, *N*_g_ > *N*_sc_^3/7^ (*N*_g_ ≈ 5.3 for *N*_sc_ = 50), the stretchability of a fBB polymer can be greater than its linear counterpart (thick line, Figure 5b). This behavior is in remarkable contrast to cBB polymers (see eq 61), ], which can never be more stretchable than their linear counterparts (thin line, Figure 5b).

One can estimate the range of extensibility affordable by fBB polymers. As the spacer segment size increases *N*_g_ = 1 to *N*_g,K_ ≈ 7.5, the extensibility increases by nearly 9 times. Thus, using fBB polymers as network strands is expected to result in extremely stretchable networks. For instance, for a fBB polymer consisting of 200 PDMS side chains of MW 5 kDa and PMMA backbone ([*N*_sc_, *n*_sc_] of [200, 68]), at *N*_g_ = *N*_g,K_ ≈ 7.5 the network becomes extremely stretchable with λ_max_^BB^ ≈ 30. Considering that the MW of the side chains is much higher than that of the spacer segment (*N*_sc_ ≫ *N*_g_), the MW of the fBB polymer is dominated by the side chains. Thus, the shear modulus of the fBB polymer network is nearly constant, which is about *k*_B_*T* per volume of the fBB polymer as the corresponding networks are unentangled. Consequently, using fBB polymers as network strands would allow for decoupling stiffness-extensibility trade-off in single-network elastomers, the fundamental component of all kinds of polymer networks.^52^

## 6 Comparison between Theory and Experiments

### 6.1 Interbackbone Distance

**Figure 6:**
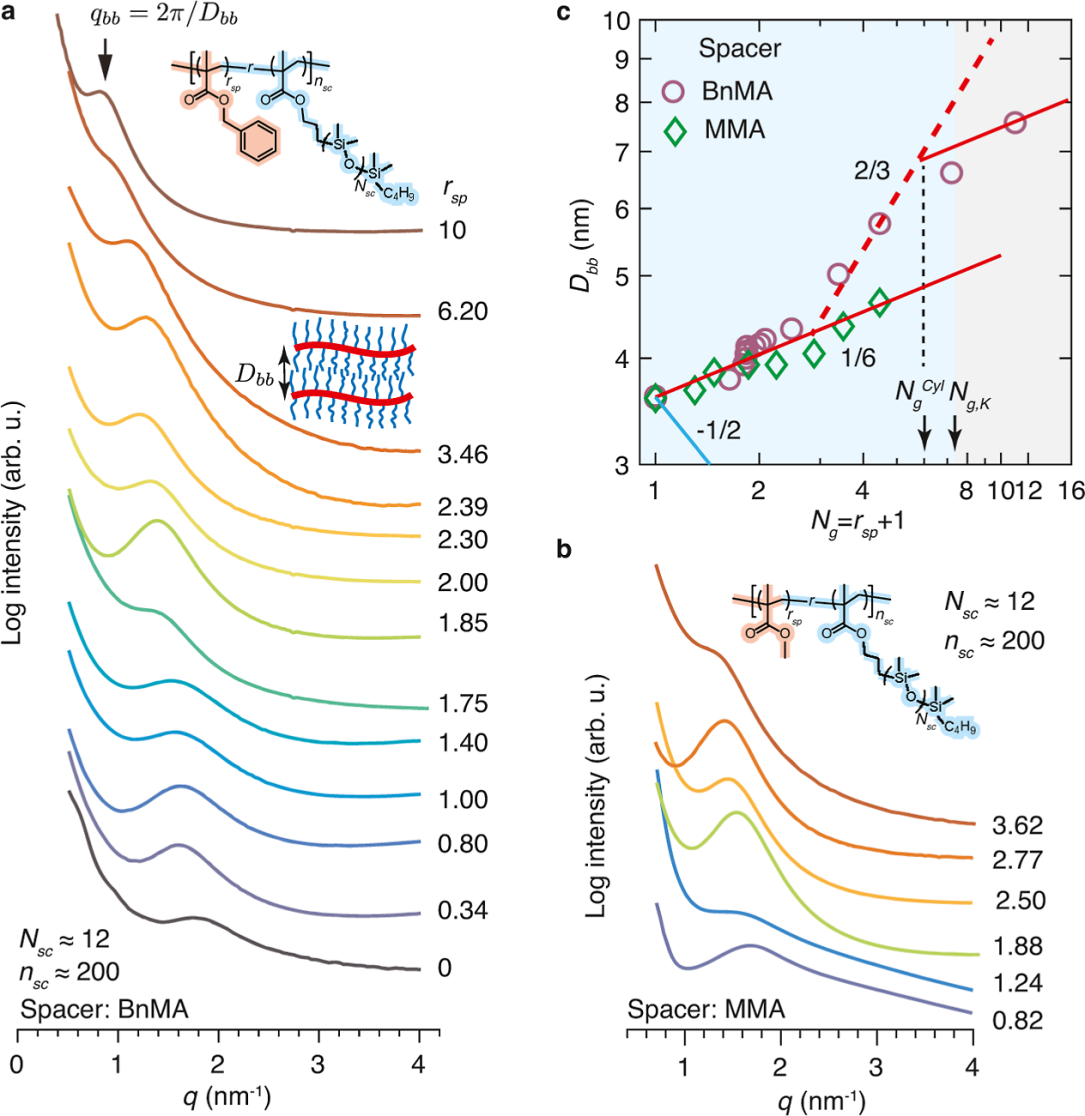
Experimentally measured interbackbone distance of foldable bottlebrush polymers in the melt. (a) Representative wide-angle X-ray scattering (WAXS) profiles of grafted polymers consisting of PDMS side chains spaced by BnMA monomers. For all grafted polymers, the number of side chains (*n*_sc_) is fixed at approximately 200, whereas the spacer/side chain ratio, *r*_sp_, increases from 0 to 10. This corresponds to the average spacer segment DP, *N*_g_ = *r*_sp_ + 1, up to 11; this value is larger than *N*_g_^**^ ≈ 1.5 (eq 19) and *N*_g_^*^ ≈ 3.7 (eq 21) for cBB polymers and *N*_g,K_ ≈ 7.5 (eq 41) for foldable bottlebrush polymers. The interbackbone distance is given by *D*_bb_ = 2π/*q*_bb_, in which *q*_bb_ is the wavenumber of the scattering peak. (b) WAXS profiles of grafted polymers consisting of PDMS side chains spaced by MMA monomers. (c) Dependence of interbackbone distance (*D*_bb_) on the average spacer segment DP *N*_g_ for two kinds of spacer monomers. Circles: BnMA spacer; diamonds: MMA spacer. Solid blue line: The prediction for cBB polymers assuming the backbone and side chains are incompatible, *D*_bb_ ∝ *N*_g_^–1/2^ for *N*_g_ < *N*_g_^**^ (eq 59). Red lines: The prediction for fBB polymers with *N*_g_ < *N*_g,K_, *D*_bb_ ∝ *N*_sc_^1/2^*N*_g_^1/6^ + *N*_g_^2/3^ (eqs 64 and 67). *N*_g,K_, the upper limit of Regime III (thin cylinder) (eq 41). *N*_g_^cyl^, the average DP of the spacer segment above which the diameter of the cylindrical core is greater than the size of the side chain (eq 73).

In our previous work, we synthesized two series of bottlebrush polymers with PDMS side chains.^51,52^ We fixed the MW of the PDMS side chains at 1000 g/mol (*N*_sc_ ≈ 12 excluding the methyl methacrylate functional group) and the number of side chains at approximately 200 (*n*_sc_ ≈ 200), while increasing only the average spacer segment DP *N*_g_. Moreover, we used two kinds of spacer monomers, BnMA and MMA, both of which are highly incompatible with PDMS with χ > 0.1. The contrast in electron density between methacrylate-based bottlebrush backbone and PDMS side chains allows the interbackbone distance to be unambiguously quantified using WAXS measurements, as shown by the representative scattering profiles in Figure 6a. In the present work, we synthesize additional bottlebrush polymers to expand the range of *N*_g_ from 1 to 11 for BnMA spacer and from 1 to 4.7 for MMA spacer (see Supporting Information). These two series of samples allow us to test the theoretical predictions of interbackbone distance for fBB polymers in the melt.

The interbackbone distance for the bottlebrush polymer without spacers (*N*_g_ = 1) is 3.6 nm (Figure 6a). This value agrees with that reported by another laboratory for the same bottlebrush polymer.^14^ Moreover, the experimentally measured value agrees well with the theoretical prediction: *D*_bb,e_ = α(2*R*_sc_), in which  nm (eq 17) and α = 0.75 is the scaling perfector. These results confirm that the bottlebrush diameter can be measured as interbackbone distance *D*_bb_ using WAXS.

As the average spacer segment DP *N*_g_ increases from 1 to 1.83, the interbackbone distance *D*_bb,e_ increases by nearly 10% from 3.6 to 4.0 nm (dark blue squares, Figure S1). Yet, for bottlebrush polymers of nearly the same spacer segment DP (*N*_g_ = 1.83) but various number of side chains (*n*_sc_ from 200 to 534), the location of the scattering peaks is nearly the same, as shown in Figure S1. These experimental results show that the interbackbone distance is independent of the number of side chains per bottlebrush, which is consistent with the theoretical prediction (eq 64).

Remarkably, the interbackbone distance increases monotonically with the decrease of grafting density for both BnMA and MMA spacers, as respectively shown by the shift of WAXS scattering peaks to lower values at higher spacer ratios in Figure 6a,b. This behavior is qualitatively different from cBB polymers in the melt, the interbackbone of which decreases at lower grafting density (higher *N*_g_) (eq 59) (blue line, Figure 6c) Moreover, for bottlebrush polymers with MMA spacer monomers, *D*_bb_ increases with *N*_g_ by a power of 1/6; this scaling relation agrees very well with the theory for fBB polymers (eq 64), as shown by the solid red line in Figure 6c.

Interestingly, for bottlebrush polymers with BnMA spacer monomers, the dependence of *D*_bb_ on *N*_g_ appears to exhibit two regimes. At relatively low spacer ratios (*N*_g_ < 3), *D*_bb_ scales with *N*_g_ by a power of 1/6. However, at relatively high spacer ratios (*N*_g_ > 3), *D*_bb_ scales with the *N*_g_ by a power between 1/6 and 2/3 (Figure 6c). This is likely because for this bottlebrush system the window for the scaling regime, *D*_bb_ ∝ *N*_g_^1/6^, is very small. Specifically, considering the difference in polymer species between the backbone and the side chains, the interbackbone distance is (eq 64)
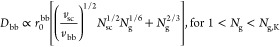
72

This expression suggests that the contribution to the interbackbone distance from the collapsed cylindrical core dominates if the spacer segment DP is greater than *N*_g_^cyl^
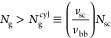
73

Typically, this condition cannot be met as the DP of side chains is much larger than that of the spacer segment (*N*_sc_ ≫ *N*_g_); moreover, the volume of a side chain monomer is comparable to that of the backbone monomer (*v*_sc_ ≈ *v*_bb_). However, for the bottlebrush polymers explored in our study, the PDMS side chain is relatively short with *N*_sc_ ≈ 12 and the ratio between the volume of a side chain monomer and that of a backbone monomer is small with *v*_sc_/*v*_bb_ ≈ 0.5 (Table 1). This gives *N*_g_^cyl^ ≈ 6, which is within the range of values (*N*_g_ from 1 to 11) explored in our study (dashed vertical line in Figure 6c). Moreover, when the diameter of the cylindrical core becomes noticeable at relatively large *N*_g_, the approximation in eq 48 becomes inappropriate. Such a correction to the side chain size is beyond the scope of this work and will be the subject of future explorations. Additionally, it would be interesting to explore whether the scaling relation recovers the 1/6 power law at very high *N*_g_ ≫ *N*_g,K_ (see eq 67). Nevertheless, *D*_bb_ is expected to increase with *N*_g_ but with an exponent intermediate between 1/6 and 2/3 near the crossover grafting densities (*N*_g_ ≈ *N*_g_^cyl^ for short side chains or *N*_g_ ≈ *N*_g,K_ for long side chains).

### 6.2 Extensibility of End-Cross-Linked Foldable Bottlebrush Polymer Networks

**Figure 7:**
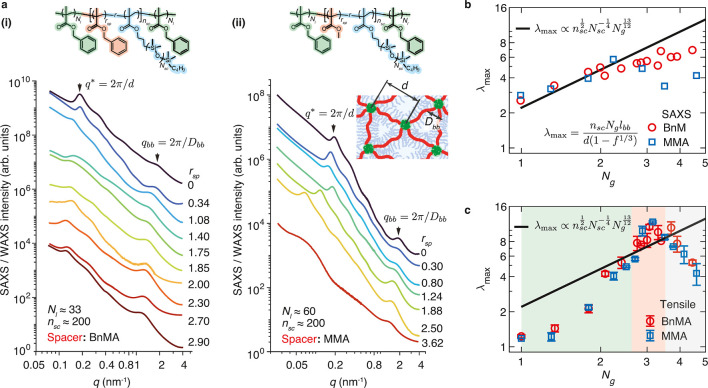
Experimentally measured extensibility of end-cross-linked foldable bottlebrush polymer networks. (a) SAXS/WAXS profiles of grafted polymers consisting of PDMS side chains spaced by (i) BnMA and (ii) MMA monomers. Inset: a schematic illustrating the microstructure of networks self-assembled by linear-fBB-linear triblock copolymers. *d*, average distance between neighboring spherical nodules aggregated by linear end blocks; *D*_bb_, average interbackbone distance. (b) Calculated network extensibility λ_max_ based on the contour length and end-to-end distance of fBB polymer network strands, which are determined by chemical synthesis and SAXS scattering. (c) Comparison between theoretical prediction and network extensibility experimentally measured by tensile tests. All measurements are performed at a constant strain rate of 0.02/s at room temperature.

It is challenging to directly quantify the size of a fBB polymer in the melt because of the limited contrast between probe molecules and the surrounding environment.^51,66^ However, it is possible to test the theoretical prediction by using fBB polymers to create linear-fBB-linear triblock copolymers that self-assemble to end-cross-linked networks (Figure 7a). In the self-assembled networks, the linear end blocks aggregate to spherical glassy nodules, which effectively cross-link the fBB polymer network strands (inset, Figure 7a). The average center-to-center domain distance, *d*, can be measured using small-angle X-ray scattering (SAXS): *d* = 2π/q^*^, where q^*^ is wavenumber of the primary scattering peak (Figure 7a). The bridging distance between two neighboring spheres, or the end-to-end distance of the fBB polymer, can be calculated as
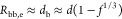
74

The contour length of the fBB polymer, *L*_max_^bb^ = *n*_sc_*N*_g_*l*_bb_ (eq 4), is determined by the molecular architecture parameters, which are prescribed through controlled chemical synthesis. Thus, it is possible to calculate the theoretical limit for the stretchability of end-cross-linked bottlebrush networks
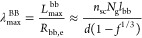
75

In our previous work, we synthesized two series of linear-fBB-linear triblock copolymers with two kinds of spacer monomers, BnMA and MMA.^52^ For each series of triblock copolymers, we fixed the number of PDMS side chains (*n*_sc_ ≈ 200) and the DP of the linear end blocks (*N*_*l*_). All these triblock copolymers are self-assembled to a random spherical microstructure, as confirmed by X-ray scattering measurements in Figure 7a. Moreover, among the four molecular architecture parameters of the linear-fBB-linear triblock copolymer, [*n*_sc_, *N*_sc_, *N*_*l*_, *N*_g_], in which *N*_*l*_ is the DP of a linear end block, three parameters are fixed and with only *N*_g_ being changed: [200, 12, 33, *N*_g_ from 1 to 4.4] for BnMA spacer monomers and [200, 12, 60, *N*_g_ from 1 to 4.6] for MMA spacer monomers. Further, WAXS measurements confirmed that the interfacial repulsion between the bottlebrush backbone and glassy nodules does not impact interbackbone distance (*D*_bb_) and that fBB polymers remain folded in self-assembled networks.^52^

Using the interdomain distance (*d*) measured by SAXS and bottlebrush contour length (*L*_max_^bb^) determined from synthesis, we calculate the network stretchability using eq 75, as shown by the symbols in Figure 7b. Despite some deviations at relatively high *N*_g_ values, when the primary scattering peaks become less pronounced (Figure 7a), the dependence of the calculated λ_max_^BB^ on *N*_g_ agrees well with the theoretical prediction, λ_max_^BB^ ∝ *N*_g_^13/12^, as shown by the solid line in Figure 7b.

Interestingly, the calculated network extensibility based on the network microstructure does not fully agree with that experimentally measured using uniaxial tensile tests (Figure 7c). For small *N*_g_ < *N*_g,*l*_ ≈ 2.8, the measured λ_max_^BB^ is lower than the calculated values. This phenomenon is commonly observed in single-network polymers. During deformation, the relatively short network strands tend to break first, leading to premature network failure.^67^ As a result, the experimentally measured network extensibility is often lower than the upper limit predicted by theory. Surprisingly, within a small window, *N*_g_∈(*N*_g,*l*_, *N*_g,*h*_ ≈ 3.4), the measured λmaxBB is higher than the calculated values. This deviation is likely due to pulling out the end linear blocks from the glassy domains.^52^

**Figure 8:**
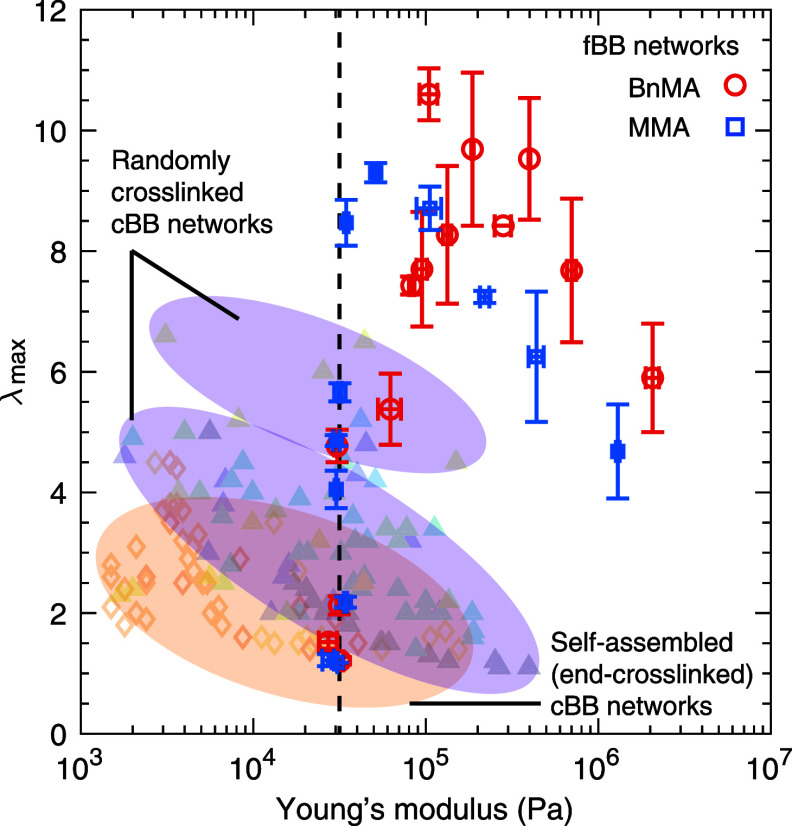
Comparison between foldable and conventional bottlebrush polymer networks. Foldable bottlebrush (fBB) polymer networks allow for truly decoupled stiffness and extensibility (vertical dashed line). By contrast, the stiffness and extensibility of conventional bottlebrush (cBB) polymer networks are negatively correlated. Because the backbone is strained by the steric repulsion among overlapped side chains, cBB polymer networks are often brittle with low extensibility unless using bottlebrush network strands of extremely high MW, when the networks become very soft with Young’s modulus on the order 1 kPa. By contrast, fBB polymer networks can exhibit remarkable extensibility while maintaining constant stiffness at relatively low spacer ratios (symbols along the dashed line). At relatively high spacer ratios, the stiffness of fBB polymers increases markedly because the glass transition temperature of fBB polymer becomes close to and even higher than room temperature (symbols on the right of the dashed line). Nevertheless, fBB polymer networks are far more stretchable than cBB polymer networks.

At a relatively high spacer ratio (*N*_g_ > *N*_g,*h*_), the experimentally measured network stretchability is lower than the limit calculated based on the network microstructure. This deviation is likely because of enhanced intramolecular interactions within the collapsed backbone of a fBB polymer. For *N*_g_ > *N*_g,*h*_, the glass transition temperature *T*_g_ of fBB polymers becomes higher than room temperature. Even without being cross-linked to form a network, at room temperature, the fBB polymer melts become stiff with modulus increases exponentially with the spacer ratio. Consequently, the stiffness of the self-assembled fBB polymer networks becomes much higher than elastic contribution of fBB polymers (*k*_B_*T* per volume of a network strand), as shown by the data points on the right of the vertical dashed line in Figure 8. Reminiscent of reduced extensibility observed in polymers near their glass transition temperature, the extensibility of fBB polymer networks is lower than the theoretical prediction, which does not account for the effects of glass transition on polymer stretchability. Nevertheless, there exists a regime in which the network modulus remains nearly constant, while the network extensibility increases markedly with the spacer ratio, as shown by the symbols along the vertical dashed line in Figure 8. By contrast, for cBB polymer networks,^12,14,16,17,38,68−72^ the network modulus is always negatively correlated to the extensibility—in other words, stiffer networks are less stretchable, as shown by the shadowed regions in Figure 8. To achieve a stretchability with λ_max_ ≈ 5, the typical limit of entangled linear polymer networks,^38^ cBB polymer networks must be extremely soft (with Young’s modulus of ∼1 kPa) (Figure 8). However, for fBB polymers with similar stretchability, their modulus can be nearly 3 orders of magnitude higher (>1 MPa) (Figure 8). Collectively, these results demonstrate that fBB polymers offer a platform for the development of polymers and soft materials of superior mechanical properties.

## 7 Conclusion and Outlook

We have developed a scaling theory to describe the molecular structure of fBB polymers in the melt. Compared to the theory for cBB polymers, where the side chains and the backbone are assumed to be compatible (χ = 0), our theory for fBB polymers considers the fact that in most cases the backbone and side chains are different chemical species and incompatible (χ > 0). We focus on fBB polymers consisting of *highly* incompatible backbone and side chains (e.g., χ > 0.1), such that the interface between distinct domains is sharp. We have identified *three* regimes depending on the side chain grafting density (1/*N*_g_).

1. Regime I. Low grafting density: Sphere (*N*_g_ > *N*_g,c_) (eq 32). The backbones of multiple grafted polymers aggregate to form a spherical core with grafting sides located at the sphere surface. The spacer segment is stretched to balance the interfacial tension between the distinct side chain and backbone domains.
2. Regime II. Intermediate grafting density: thick cylinder  (eq 41). The backbone collapses to a thick cylinder with the spacer segment being stretched to cross the cylinder cross-section to balance interfacial tension. Yet, the side chains do not experience crowding and are not stretched.
3. Regime III. High grafting density: thin cylinder . The backbone collapses into a slim cylinder with its surface densely grafted with many side chains that are radially stretched away from the backbone.

The most interesting regime for fBB polymers is associated with high grafting density of side chains (Regime III). For cBB polymers, the regimes of interest are associated with intermediate and high side chain grafting densities (Regimes III and IV). In these regimes, the grafted polymers adopt a bottlebrush-like conformation, where the side chains experience steric repulsion and are stretched or the side chains from the same grafted polymer are sufficient to occupy the volume near the backbone.

There are three major differences between fBB and cBB polymers (Table 2). First, for a fBB, the cross-section size of the collapsed cylindrical core increases with *N*_g_ by a power of 2/3, *r*_c_ ∝ *N*_g_^2/3^ (eqs 38 and 55). By contrast, for a cBB polymer the backbone is prestretched with the cross-section dimension being a constant of the backbone Kuhn segment size.

**Table 2:** Polymer Physics Parameters of Conventional and Foldable Bottlebrush Polymers^a^

Polymer type	Conventional bottlebrush	Foldable bottlebrush	Linear polymer
Design parameters	[*N*_sc_, *n*_sc_, *N*_g_]	[*N*_sc_, *n*_sc_, *N*_g_, χ]	*N* = *n*_sc_*N*_g_
Diameter, *D*_bb_	*N*_sc_^1/2^*N*_g_^–1/2^	*N*_sc_^1/2^*N*_g_^1/6^ + *N*_g_^2/3^	*b*
End-to-end distance, *R*_bb_	*n*_sc_^1/2^*N*_sc_^1/4^*N*_g_^1/4^	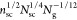	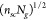
Stretchability, λ_max_^BB^	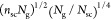	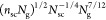	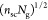
Stretching ratio, β	*N*_sc_^–1/4^*N*_g_^1/4^	*N*_sc_^–1/4^*N*_g_^7/12^	1

a A cBB polymer has three molecular architecture design parameters, [*N*_sc_, *n*_sc_, *N*_g_]. By contrast, the theory for fBB polymers considers the incompatibility between side chains and backbone; therefore, there are four design parameters, [*N*_sc_, *n*_sc_, *N*_g_, χ]. The diameter, end-to-end distance, and stretchability of a cBB polymer are given by eqs 59, 60, and 62; for a fBB polymers, they are given by eqs 64, 68, and 70, respectively. These polymer physics parameters are also compared to the linear counterpart with the same DP (*N* = *n*_sc_*N*_g_). The stretching ratio, β ≡ λ_max_^BB^/λ_max_^0^, is the extensibility of a bottlebrush polymer relative to its linear counterpart (eq 63 for cBB polymers, and eq 71 for fBB polymers).

Second, in a fBB polymer, the size of side chain *R*_sc_ increases with *N*_g_ by a power of 1/6, *R*_sc_ ∝ *N*_sc_^1/2^*N*_g_^1/6^. This behavior is qualitatively different from the case for cBB polymers, where the side chain size decreases with *N*_g_ by a power of −1/2, *R*_sc_ ∝ *N*_sc_^1/2^*N*_g_^–1/2^. Such a remarkable difference originates from the high incompatibility between the side chains and backbone polymer, which drives the backbone polymer to fold along its contour. The collapse of the backbone polymer further increases the grafting density of side chains, and therefore, results in more severe crowding of side chains, such that side chains are more extended. Consequently, the diameter, or the cross-section, of a fBB polymer increases with *N*_g_: *D*_bb_ ∝ *N*_sc_^1/2^*N*_g_^1/6^ + *N*_g_^2/3^.

Third, the size of a fBB polymer decreases at lower grafting density (or higher *N*_g_): . By contrast, for a cBB polymer, its size increases at lower grafting density: *R*_bb_ ∝ *N*_g_^1/4^. This qualitative difference has profound implications: Unlike a cBB polymer whose backbone is prestrained, a fBB polymer stores lengths in its collapsed backbone. These stored lengths can be released upon large strain, enabling remarkable stretchability.^52^

The predictions of our theory on the dependencies of bottlebrush diameter and size on grafting density (1/*N*_g_) have been partially verified by experiments. However, it remains to be systematically tested the dependencies of bottlebrush diameter and size on all four molecular architecture parameters [*N*_sc_, *n*_sc_, *N*_g_, χ]. Of particular interest is the crossover between thin and thick cylinders (Regimes II and III), which requires bottlebrush polymers with relatively high side chain MW and high spacer ratios (large *N*_sc_ and *N*_g_).

It should be noted that our theory assumes no mixing between the backbone and side chains at the molecular level. Yet, decades of research in block copolymer self-assembly have shown that, even for highly incompatible polymers, there is some extent of interfacial mixing, despite that the thickness of the interfacial layer becomes comparable to monomer size for strongly segregated block copolymers.^56,57^ At low grafting densities, the microphase separation involves the aggregation of multiple grafted polymers. By contrast, at high grafting densities, when the backbone is completely shielded by the side chains, the collapse of the backbone occurs within individual grafted polymers. Although the concept of segregation strength from classical block copolymer self-assembly can be extended to describe the onset of microphase separation within a grafted polymer, it applies only to regimes with relatively low grafting densities of less than one side chain per Kuhn segment (*N*_g_ > *N*_K_, eq 46 in Regime II). It remains an open question whether similar approaches apply to grafted polymers with high grafting densities (*N*_g_ < *N*_K_, Regime III).

A bottlebrush polymer is often treated as a ‘fat’ linear polymer with effective Kuhn length on the scale of the bottlebrush cross-section size. However, the effective Kuhn segment is not necessarily isotropic. Unlike a classical semiflexible linear biopolymer, which is typically incompressible along its cross-section, in a semiflexible bottlebrush polymer the side chains are compressible. As a result, the effective Kuhn segment is anisotropic: it exhibits worm-like chain (WLC) behavior along the backbone but is compressible along its cross-section. Accounting for the anisotropy in the effective Kuhn segment does not affect our theory for the extensibility of bottlebrush polymers but would be critical to understanding the behavior of bottlebrush polymer networks under compression.

Our theory does not account for the effects of intramolecular interactions on the physical properties of fBB polymers. For instance, our experiments show that at relatively high spacer ratios, the *T*_g_ of the collapsed bottlebrush backbone approaches room temperature,^52^ indicating enhanced intramolecular interactions. As a result, the modulus of fBB polymer networks can be dramatically increased using high *T*_g_ spacer monomers to reach MPa (Figure 8) without substantially compromising network extensibility (Figure 7c). By contrast, the modulus of cBB polymer networks is often much lower than ∼1 MPa, the entanglement modulus of their linear counterpart.^13^ These findings indicate that of the feasibility of applying the concept of fBB polymer networks to create structural polymers with high modulus and high extensibility.

Our experimental systems are bottlebrush polymers with methacrylate-based backbones, which are flexible linear polymers. Alternatively, bottlebrush polymers with a norbornene-based backbone have been extensively studied, largely because they can be synthesized in a controllable and relatively straightforward manner.^3^ However, based on our experience, the intrinsic chain rigidity^73^ prevents norbornene-based bottlebrush polymers from folding. Nevertheless, the question of how backbone rigidity affects the molecular structure of foldable bottlebrush polymers remains open. Finally, by accounting for polymer swelling, the theory for the molecular structure of fBB polymers in the melt can be readily extended to bottlebrush polymers with incompatible side chains and backbone in the presence of solvents. This could provide foundational insights into the use of fBB polymers as building blocks for creating molecular-architecture-encoded biomaterials.

## Data Availability

All data are available in the manuscript or the Supporting Information.

## Supporting Information Available

WAXS data, GPC profiles, and ^1^H NMR spectra (PDF)

## Abbreviation

sc: side chain of a grafted polymer
bb: backbone of a grafted polymer
*C*_*∞*_: Flory’s characteristic ratio
*θ*: bond angle
*m*_0_: mass of a chemical monomer
*M*_0_: mass of a Kuhn segment
*ρ*: polymer density
*χ*: Flory–Huggins interaction parameter
*l*: length of all main-chain bonds of a chemical monomer
*l*_0_: length of one main-chain bond
*v*: volume of a chemical monomer
*v*_K_: volume of a Kuhn segment
*b*: length of a Kuhn segment
*d*: average center-to-center distance between two neighboring spherical domains in a network self-assembled by linear-fBB-linear triblock copolymers
*d*_b_: bridging distance between two neighboring spherical domains, eq 74
*D*_bb_: interbackbone distance between neighboring bottlebrush polymers in the melt, eqs 59, 64, and 72
*f*: composition of a grafted polymer or a block copolymer, eqs 45, 74, and 75
*F*_tot_: total free energy of a grafted polymer, eqs 22, 28, 37, 39, 53, and 54
*F*_int_: interfacial free energy between the incompatible backbone and side chains, eqs 24, 33, and 35
*F*_sc_: entropic free energy due to the stretching of the side chains, eq 50
*F*_bb_: entropic free energy due to the stretching or confining the backbone polymer, eqs 27, 36, and 52
*F*_sp_^cly^: free energy of a spacer segment and a side chain of a fBB polymer, eq 44
*F*_sp_^dis^: free energy of a spacer segment and a side chain in the disordered state, eq 45
*F*_c_: confinement free energy of the backbone of a fBB polymer, eq 51
*g*: number of monomers for a section of the backbone polymer passing through the side chain pervaded volume for cBB polymers
*g*_t_: number of monomers per tension blob, eq 12
*Q*^*^: number of grafted polymers per micelle, eq 29
*n*_sc_: number of side chains per grafted polymer
*N*: degree of polymerization (DP)
*N*_l_: DP of a linear end block in linear-fBB-linear triblock copolymers
*N*_g_: average DP of a spacer segment between two neighboring grafting sites
*N*_K_: DP per Kuhn segment
*N*_sc_: DP of a side chain
*N*_bb_: DP of the backbone of a grafted polymer
*N*_g_^*^: crossover spacer segment DP (equivalent to grafting density 1/*N*_g_^*^), below which a conventional grafted polymer adopts a bottlebrush molecular structure, eqs 6, 20 and 21
*N*_g_^**^: crossover spacer segment DP below which a conventional grafted polymer becomes a dense bottlebrush, eqs 9, and 19)
*N*_g,c_: crossover spacer segment DP at which there is only one grafted polymer per micelle, eq 32
*N*_g,K_: crossover spacer segment DP above which the backbone collapses to a thick cylinder with the spacer segment being stretched to across the cylinder cross-section, eq 41
onset of segregation strength above which microphase separation occurs within a grafted polymer, eq 46
*N*_g_^cyl^: crossover spacer segment DP which the cross-section of collapsed backbone is greater than side chain size in a fBB polymer, eq 73
*h*_sc_: shell thickness of a fBB polymer, eq 66
persistence length of bottlebrush, eq 18
*L*_c_: contour length of the cylindrical core in a fBB polymer, eq 34
*L*_c,e_: equilibrium contour length of the cylindrical core in a fBB polymer, eq 68
*L*_max_: polymer contour length, eqs 2, and 4
*p*: packing ratio, eq 43
*P*_sc_: number of side chains within pervaded volume *V*_p_, eq 8
*q*^*^: the magnitude of the wavevector associated with the primary scattering peak
*r*_0_: length scale determined by the Flory–Huggins interaction parameter χ and the polymer physics parameters of the backbone of fBB polymer, eq 30
*r*_c,e_: equilibrium cross-section size of the domain collapsed by bottlebrush backbone, eqs 23, 31, 38, 55
*r*_g,0_: unperturbed size of a spacer segment, eq 26
*R*_bb,0_: unperturbed size of bottlebrush backbone, eq 3
*R*_bb_: size of bottlebrush backbone, eqs 16, 18, 60, and 69
*R*_bb,e_: equilibrium size of a fBB polymer, eqs 68, and 69
*R*_sc_: size of a side chain, eqs 17, 47, 48, 49, and 56
*R*_sc,0_: unperturbed size of a side chain, eq 1, and 11
*R*_sc,e_: equilibrium side chain size in fBB polymer, eq 56
*s*_sc_: packing parameter associated with the ratio of the volume of a chemical monomer to the volume of a rod-like Kuhn segment, eq 10
*V*_p_: pervade volume by an unperturbed side chain
*z*: ratio between the length and volume of a Kuhn segment
*β*: stretching ratio of a bottlebrush polymer compared to its linear counterpart, eqs 58, 63, and 71
*γ*: polymer–polymer interfacial tension, eq 25
*λ*_max_^0^: maximum extensibility of a linear polymer, eq 58
*λ*_max_^BB^: maximum extensibility of a bottlebrush polymer, eqs 57, and 62
*ξ*_t_: size of a tension blob, eqs 13, and 40
