# Supplementary material for: Molecular Structure of Foldable Bottlebrush Polymers in Melts
Source: Macromolecules. 2025 Apr 1;58(8):4320–39. doi: 10.1021/acs.macromol.4c02981 (PMC12020426; doi:10.1021/acs.macromol.4c02981)
Supplement: Supplementary file 1 — ma4c02981_si_001.pdf [file ma4c02981_si_001.pdf]

# Supporting Information

## Molecular structure of foldable bottlebrush polymers in melts

Li-Heng Cai[1,2,3,4]*

**Affiliations:**

[1]Soft Biomatter Laboratory, Department of Materials Science and Engineering, University of Virginia, Charlottesville, VA 22904, USA

[2]Department of Chemical Engineering, University of Virginia, Charlottesville, VA 22904, USA

[3]Department of Biomedical Engineering, University of Virginia, Charlottesville, VA 22904, USA

[4]Department of Chemistry, University of Virginia, Charlottesville, VA 22904, USA

*Corresponding author. Email: liheng.cai@virginia.edu

**The PDF file includes:**

SI Text
Figure S1
Supplementary Data

**SI Text**

**Micelles with side chains as the core.** At very low grafting densities, multiple grafted polymers may aggregate to form a micelle with side chains as the core. The radius of the spherical domain, $r_c$, is determined by the mass conservation of the side chains:

$$r_c \approx \left(\frac{3}{4\pi} Q n_{sc} N_{sc} v_{sc}\right)^{1/3} \propto (Q n_{sc} N_{sc})^{1/3} \quad \text{(S1)}$$

Here, the aggregation number $Q$ corresponds to the number of grafted polymers per micelle. The interfacial free energy of the spherical domain is proportional to the surface area of the sphere:

$$F_{int}^{m} \approx 4\pi r_c^2 \gamma \propto \gamma (Q n_{sc} N_{sc})^{2/3} \quad \text{(S2)}$$

Within the spherical domain, each side chain is stretched from its unperturbed size, $R_{sc,0}$, to the size of the spherical domain, $r_c$, to ensure that all grafting sites are at the surface of the spherical domain.

$$R_{sc,0} \approx (N_{sc} b_{sc} l_{sc})^{1/2} = (b l N_{sc})^{\frac{1}{2}} \quad \text{(S3)}$$

Since there are $n_{sc}$ side chains per grafted polymer, the entropic free energy attributed to stretching the backbone of one grafted polymer is:

$$F_{sc} \approx k_B T n_{sc} \frac{r_c^2}{R_{sc,0}^2} \quad \text{(S4)}$$

The total free energy of an individual grafted polymer within the micelle is:

$$F_{tot} = \frac{F_{int}^{m}}{Q} + F_{sc} \approx \left(\frac{3}{4\pi} n_{sc} N_{sc} v_{sc}\right)^{\frac{2}{3}} \left[4\pi\gamma Q^{-\frac{1}{3}} + k_B T \frac{n_{sc}}{b_{sc} l_{sc} N_{sc}} Q^{\frac{2}{3}}\right] \tag{S5}$$

Minimizing the free energy gives the equilibrium aggregation number $Q^*$:

$$Q^* \approx 4\pi \frac{\gamma b_{sc} l_{sc}}{k_B T} \frac{N_{sc}}{n_{sc}} \approx 4\pi \frac{(r_0^{sc})^3}{v_{sc}} \frac{N_{sc}}{n_{sc}} \propto \frac{N_{sc}}{n_{sc}} \chi^{1/2} \tag{S6}$$

Here, $r_0^{sc}$ is a length scale determined by the Flory-Huggins interaction parameter $\chi$ and the polymer physics parameters of the side chain:

$$r_0^{sc} \equiv \left(\frac{\gamma}{k_B T} v_{sc} b_{sc} l_{sc}\right)^{\frac{1}{3}} = \left(\frac{\gamma}{k_B T} vbl\right)^{\frac{1}{3}} \equiv r_0 \propto \chi^{\frac{1}{6}} \left(\frac{vl}{b}\right)^{1/3} \tag{S7}$$

Substituting eq. (S6) into eq. (S1) obtains the equilibrium size of the spherical core:

$$r_{c,e} \approx r_0^{sc} N_{sc}^{\frac{2}{3}} \propto \left(\frac{vl}{b}\right)^{1/3} \chi^{\frac{1}{6}} N_{sc}^{\frac{2}{3}} \tag{S8}$$

The above derivations are essentially the same as those for micelles with the backbone as the core except by replacing the spacer segment DP ($N_g$) with that of the side chain ($N_{sc}$) (see **Section 4.1**). Thus, for $N_g > N_{sc}$, it is energetically favorable for side chains to form the core; this would result in smaller aggregation number $Q^*$ [eq. (S6) and eq. (29)] and thus lower free energy. The opposite would occur for $N_g < N_{sc}$. Nevertheless, the aggregation number must be no less than one ($Q^* \geq 1$); this gives:

$$N_{sc} \geq n_{sc} \chi^{-\frac{1}{2}} > n_{sc} \tag{S9}$$

Equation (S9) indicates that to form micelles with the side chains as the core, the side chain size must be greater than the number of side chains per grafted polymer. However, in this paper, we restrict out consideration to $n_{sc} \gg N_{sc}$. Thus, we focus on the cases where grafted polymers form micelles with the backbone as the core.

**Supplementary Figures**

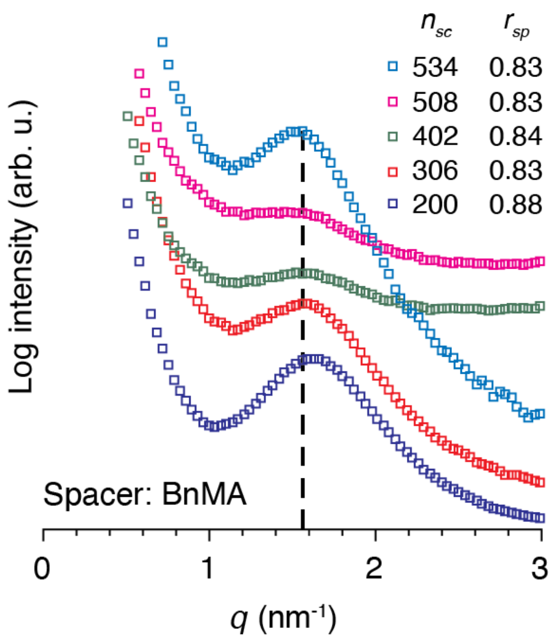


**Fig. S1. WAXS profiles of fBB polymers with various number of side chains.**

Representative wide angle X-ray scattering profiles of grafted polymers consisting of PDMS side chains spaced by BnMA monomers. All grafted polymers have nearly the same BnMA spacer/side chain ratio ($r_{sp} \approx 0.83$) but various number of side chains ($n_{sc}$ from 200 to 534).

**Supplementary Data | GPC profiles and $^1$H NMR spectra for bottlebrush polymers**

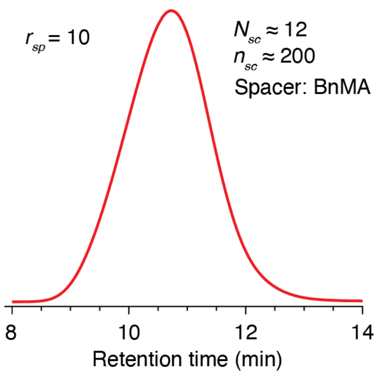


Gel permeation chromatography (GPC) trace of bottlebrush polymers with BnMA as spacer monomers. The spacer/side chain ratio is 10.

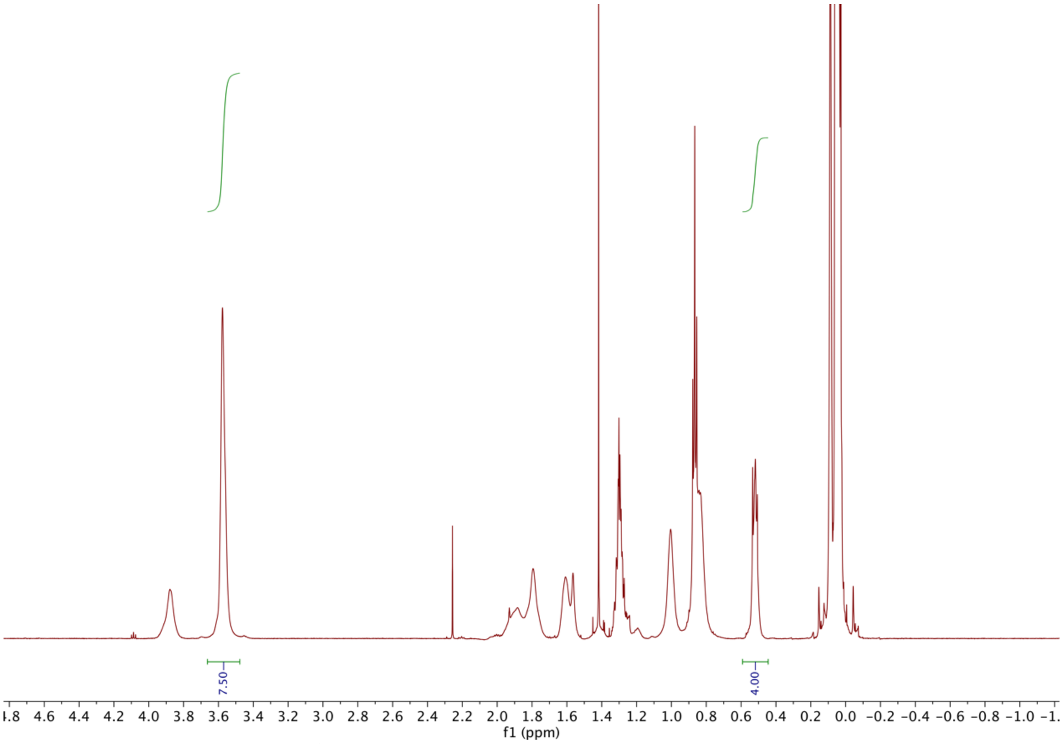


$^{1}$H NMR of (MMA$_{2.50}$-*r*-PDMS$^{1}$)$_{200}$. The number of PDMS side chains is 200, the spacer/side chain ratio $r_{sp}$ is 7.50/3=2.50, and the number of MMA monomers is 200×2.50=500.

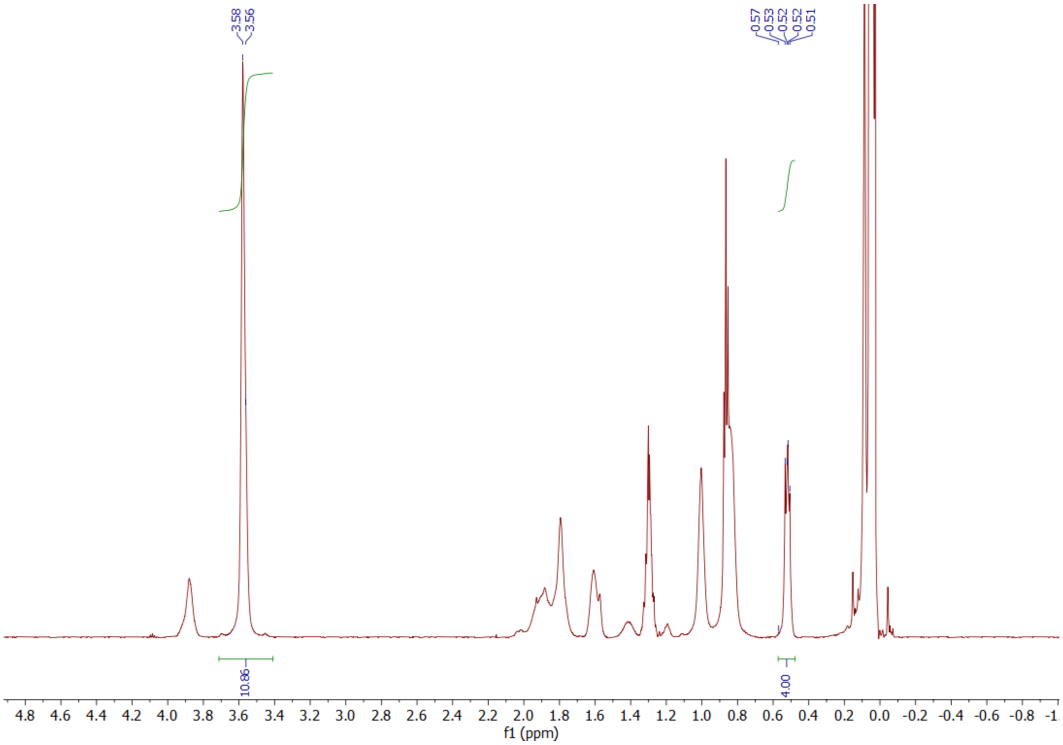


$^{1}$H NMR of (MMA$_{3.62}$-*r*-PDMS$^{1}$)$_{192}$. The number of PDMS side chains is 192, the spacer/side chain ratio $r_{sp}$ is 10.86/3=3.62, and the number of MMA monomers is 192×3.62=695.

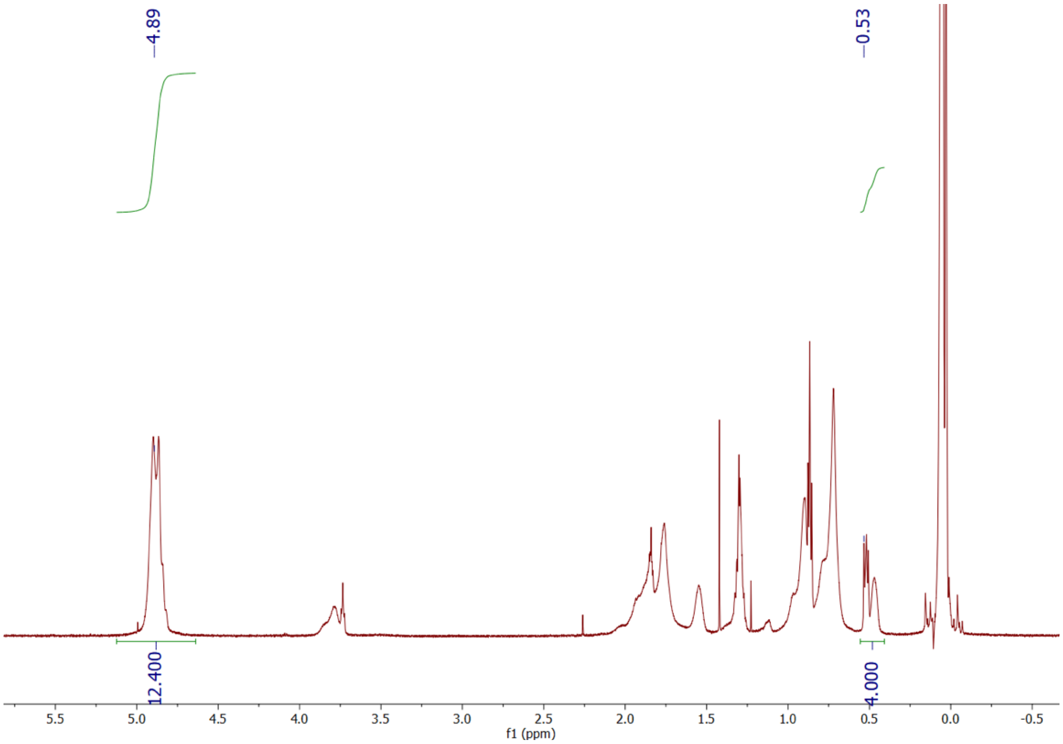


$^{1}$H NMR of (BnMA$_{6.20}$-$r$-PDMS)$_{198}$. The number of PDMS side chains is 198, the spacer/side chain ratio $r_{sp}$ is 12.400/2=6.20, and the number of BnMA monomers is 198×6.20=1228.

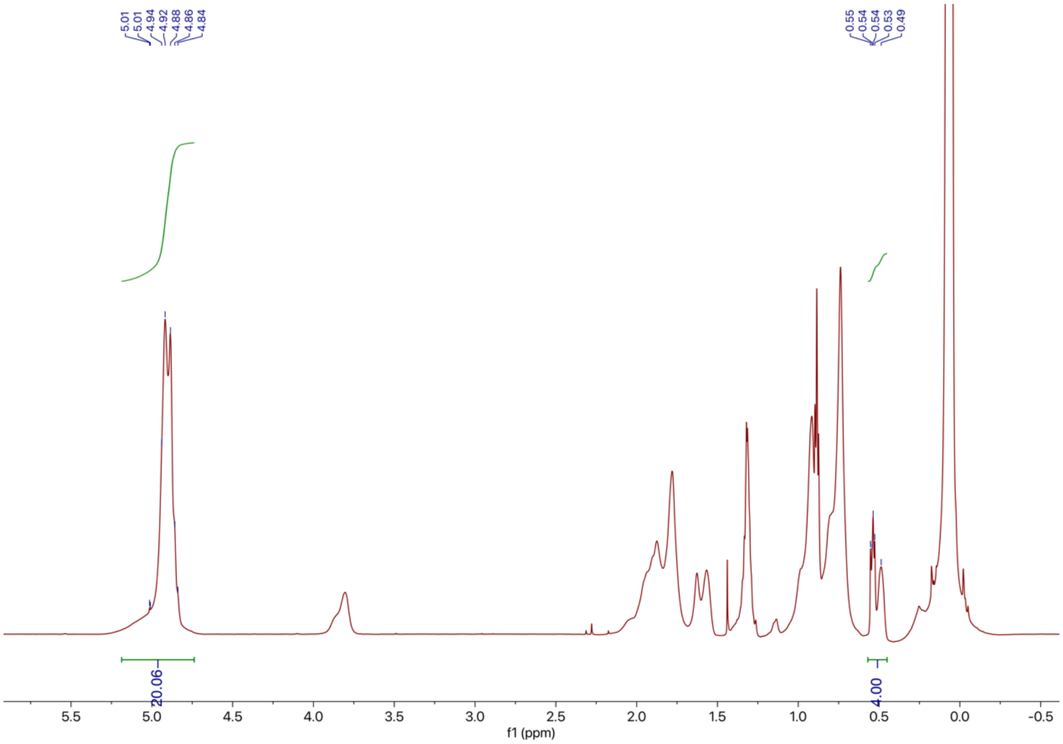


$^{1}$H NMR of (BnMA$_{10.03}$-$r$-PDMS)$_{198}$. The number of PDMS side chains is 198, the spacer/side chain ratio $r_{sp}$ is 20.06/2=10.03, and the number of BnMA monomers is 198×10.03=1986.
